# Recalibration Methods for Improved Clinical Utility of Risk Scores

**DOI:** 10.1177/0272989X211044697

**Published:** 2021-10-04

**Authors:** Anu Mishra, Robyn L. McClelland, Lurdes Y. T. Inoue, Kathleen F. Kerr

**Affiliations:** School of Public Health, Imperial College London, London, UK; Department of Biostatistics, University of Washington, Seattle, WA, USA; Department of Biostatistics, University of Washington, Seattle, WA, USA; Department of Biostatistics, University of Washington, Seattle, WA, USA

**Keywords:** calibration, recalibration, clinical utility, net benefit, risk prediction

## Abstract

**Background:**

An established risk model may demonstrate miscalibration, meaning predicted risks do not accurately capture event rates. In some instances, investigators can identify and address the cause of miscalibration. In other circumstances, it may be appropriate to recalibrate the risk model. Existing recalibration methods do not account for settings in which the risk score will be used for risk-based clinical decision making.

**Methods:**

We propose 2 new methods for risk model recalibration when the intended purpose of the risk model is to prescribe an intervention to high-risk individuals. Our measure of risk model clinical utility is standardized net benefit. The first method is a weighted strategy that prioritizes good calibration at or around the critical risk threshold. The second method uses constrained optimization to produce a recalibrated risk model with maximum possible net benefit, thereby prioritizing good calibration around the critical risk threshold. We also propose a graphical tool for assessing the potential for recalibration to improve the net benefit of a risk model. We illustrate these methods by recalibrating the American College of Cardiology (ACC)–American Heart Association (AHA) atherosclerotic cardiovascular disease (ASCVD) risk score within the Multi-Ethnic Study of Atherosclerosis (MESA) cohort.

**Results:**

New methods are implemented in the R package *ClinicalUtilityRecal*. Recalibrating the ACC-AHA-ASCVD risk score for a MESA subcohort results in higher estimated net benefit using the proposed methods compared with existing methods, with improved calibration in the most clinically impactful regions of risk.

**Conclusion:**

The proposed methods target good calibration for critical risks and can improve the net benefit of a risk model. We recommend constrained optimization when the risk model net benefit is paramount. The weighted approach can be considered when good calibration over an interval of risks is important.

Risk models can help clinicians and patients make health care decisions. Recommendations for specific interventions can be based on comparing patients’ estimated risk of a particular clinical outcome to a predefined risk threshold. In 2013, the American College of Cardiology (ACC) and the American Heart Association (AHA) published guidelines recommending that individuals with an estimated 10-y risk of atherosclerotic cardiovascular disease (ASCVD) greater than 7.5% receive statin therapy.^1^ Paired with this guideline, the panel developed the ACC-AHA-ASCVD risk calculator to estimate 10-y ASCVD risk, with recommendations to reassess risk every 4 to 6 y in adults aged 40 to 79 y free of ASCVD.

In such settings, risk model calibration carries heightened importance. The calibration of a risk model refers to the agreement between predicted risks and observed rates of events. There is evidence that the ACC-AHA-ASCVD risk calculator substantially overestimates the risk of ASCVD.^2^ Following risk-based treatment guidelines, using overestimated risks implies overtreatment in the population. Hence, miscalibration can have a serious public health impact.^3^

When an established risk model is applied to a new population, we are particularly concerned that predicted risks may not be well calibrated. In addition, a well-calibrated model may become miscalibrated over time.^4^ Ideally, when miscalibration appears, one can identify and address the cause. However, this is not always possible. Miscalibration can arise for complex reasons or because of fundamental differences between populations. In such instances, it will not be possible to eliminate the source of miscalibration.^5^ When a model is poorly calibrated and development of a new model is infeasible or undesirable, then it may be prudent to use statistical methods to recalibrate the risk model. However, existing methods for risk model recalibration do not account for how the risk model will be used in clinical practice. In this work, we propose 2 methods for risk model recalibration when the purpose of the risk model is to recommend for or against an intervention based on a predetermined risk threshold.

Before implementing recalibration methods, researchers may wish to understand whether recalibration has the potential to improve the usefulness of a risk model. We propose a graphical tool to help with this assessment. The tool indicates when specialized methods of recalibration, such as those proposed, have the potential to improve the clinical utility of a risk model beyond standard methods of recalibration.

First, we define terminology and notation and summarizes key background material. Next, we introduce a the graphical device to help researchers assess the potential for recalibration to improve the clinical utility of a risk model. Following this, we propose 2 new methods of recalibration, weighted logistic recalibration and constrained logistic recalibration. We present simulation results and illustrate the use of the graphical tool and apply the proposed methods to recalibration the ACC-AHA-ASCVD risk model within the ethnically diverse Multi-Ethnic Study of Atherosclerosis (MESA) cohort. We close with a discussion of the materials presented.

## Methods

### Preliminaries

#### Notation and definitions

Y denotes the clinical outcome (ASCVD events in the example given above). Throughout this work, we refer to individuals who experience the event without intervention as *cases* (i.e., Y=1 ) and individuals who do not experience the event (i.e., Y=0 ) as *controls*. In the population without intervention, π=P(Y=1) , we refer to π as the *prevalence* of the outcome, as is customary in the biomarker literature.

The expected benefit of the intervention to a would-be case is B . Controls expect harm or cost of the intervention C . We note that B encapsulates both the positive and negative aspects of the intervention for cases. In our application, the benefit B is the reduction of ASCVD events (due to statins) to an individual who would have an event without intervention, after accounting for the monetary costs and side effects of statins. R is the risk threshold for recommending for/against the intervention. As noted above, R=7.5% in the ASCVD example. Here and throughout, the term *risk threshold* refers to the clinically relevant threshold used to assign intervention, defined a priori. We use the term *cutpoint* to refer to any generic threshold.

Let S be a risk model for Y , based on 1 or more predictors (risk factors). We call $S_{i}$ the *predicted risk, estimated risk*, or *risk score* (equivalently) for individual i . Z=logit(S) is the logit-transformed risk score. The premise of this article is that there is an existing risk model S that we are interested in recalibrating. We assume that S is monotonically nondecreasing with risk, meaning $S_{i}\;>\;S_{j}\Rightarrow P[Y_{i}=1|S_{i}]\geq P[Y_{j}=1|S_{j}]$ . If S did not have this monotonicity property, we would likely not be interested in recalibrating it.

Here and throughout, we assume a data set is available for recalibrating the risk model S . The data set is a random sample of the relevant population with observed outcomes Y without the intervention. S′ is a recalibration of S as long as S′=f(S) for some monotone nondecreasing function f:[0,1]→[0,1] .

#### Risk model calibration

Different notions of calibration have appeared in the literature on risk models. Van Calster et al.^6^ presented 4 notions of risk model calibration: strong calibration, moderate calibration, weak or logistic calibration, and calibration-in-the-large. These types of calibration are hierarchical: strong calibration implies moderate calibration, moderate calibration implies weak calibration, and weak calibration implies calibration-in-the-large. The definition of calibration in this article is “moderate calibration,” as defined by Van Calster et al.^6^ and is formally expressed as follows. For risk model S estimating risk of binary outcome Y , S is calibrated at *r* if P[Y=1|S=r]=r . If P[Y=1|S=r]=r for all r∈[0,1] , then we say S is calibrated.

The calibration of a risk model can be assessed by examining observed event rates in groups with similar predicted risks. In Hosmer-Lemeshow plots, predicted risks are typically grouped by deciles; for each decile, the event rate for the decile is plotted against the average predicted risk in that group.^7^ Alternatively, smoothing functions (such as a LOESS smoother) can be used to generate a calibration curve.^8^ The calibration curve for a calibrated risk model is the identity line.

#### Logistic recalibration and other methods

Logistic recalibration, proposed by Cox in 1958, is the most prominent method of recalibration.^9^ Under logistic recalibration, f has the form $f=\mathrm{expit}(\alpha_{0}+\alpha_{1}\mathrm{logit}(S))$ , where $\alpha_{0}$ is the recalibration intercept and $\alpha_{1}$ is the recalibration slope. The recalibration intercept and slope, $\alpha_{0}$ and $\alpha_{1}$ , are estimated by fitting a simple linear logistic regression model in which Y is regressed on the logit-transformed risk scores Z . Recalibrated risk scores are generated by scaling Z by ${\hat{\alpha }}_{1}$ , shifting by ${\hat{\alpha }}_{0}$ , then transforming back to the risk scale via the inverse of the logit function. Note that this is a family of valid recalibration functions for any real $\alpha_{0}$ and positive $\alpha_{1}$ .

More recently, more flexible methods of recalibration have been proposed.^10–14^ The greater flexibility of such methods raises the possibility of overfitting. Some alternative methods are not guaranteed to produce a monotone transformation of the original risk score. We consider that a nonmonotone transformation fundamentally changes a risk model and should not strictly be considered a recalibration of the risk model. Some flexible methods of recalibration have been seen to perform poorly for risk models constructed using logistic regression.^15^ Although not a presented as a method of recalibration per se, the risk-mapping plot developed under the relative utility framework has potential to produce a recalibrated risk marker through similarly flexible methods, with requirements that ensure monotonicity.^16^ The goal of the approaches proposed in this article is to retain the parsimony of Cox’s logistic recalibration while prioritizing calibration near the clinically important risk threshold.

#### Clinical utility of risk models for treatment decisions based on risk

The clinical utility of a risk model refers to the usefulness of a risk model for its intended clinical application. The standardized net benefit ( sNB ) is the measure of risk model clinical utility considered in this article. Given a risk model S for outcome Y and risk threshold R for recommending an intervention to prevent or ameliorate Y ,

(1) $$\mathrm{sN}B_{R}=\mathrm{TP}R_{R}-\frac{R}{1-R}\frac{1-\pi }{\pi }\mathrm{FP}R_{R}.$$

where $\mathrm{TP}R_{R}$ ( $\mathrm{FP}R_{R}$ ) is the true-positive rate (false-positive rate) for the risk model using risk threshold R . $\mathrm{sN}B_{R}$ captures the utility of the risk model to correctly assign intervention to cases, discounted by the proportion of controls receiving intervention, where the “discounting factor” accounts for the prevalence and harms and benefits of intervention.^17–20^ Henceforth, we suppress notation showing the dependence of sNB on R . Unless stated explicitly otherwise, we presume the risk threshold R for all calculations of sNB .

A key assumption is that the risk threshold R accurately represents the benefits and harms of the intervention according to the relation $\frac{R}{1-R}=\frac{C}{B}$ .^17,20–22^ In the ASCVD example, the risk threshold R=7.5% implies the benefit ( B ) of statins to a case is about 12 times greater than the harm of statin therapy ( C ) to a control. Further, the harm-to-benefit ratio must be independent of the risk model.^20^ We adopt these assumptions throughout.

We note that we use the “opt-in” formulation of sNB , indicating that the default treatment policy without a risk model should be treat none (rather than treat all).^16,23^ This article focuses on the standardized version of net benefit ( sNB ), but methods could easily be formulated in terms of net benefit ( NB ) instead. As shown in equation (1), sNB divides NB (the net benefit of intervention less net harms) by the prevalence. The maximum value of sNB is always 1, which would occur for a risk model that perfectly discriminates ( TPR=1 , FPR=0 ).^24^ We find this theoretical maximum to be useful for gauging a risk model’s clinical utility relative to the maximum possible clinical utility. There are other measures of clinical utility in the literature (notably relative utility) we do not consider here.^16,20^

#### Calibration of a risk model and its clinical utility

Van Calster and Vickers^25^ give examples using simulated data in which miscalibration reduces the clinical utility of risk-based treatment policies. As the authors note, these results are expected because net benefit is a proper scoring rule.^26^ Baker et al.^27^ established the connection between the calibration of a risk model, the slope of its receiver-operating characteristic (ROC) curve, and the prevalence, π . Metz^28^ related ROC analyses to a cost-benefit framework for decision making. We provide an alternative presentation of the result in Baker et al.,^27^ relating the height of the calibration curve for S to the prevalence π and the slope of the ROC curve. Supplementary Material A provides the full statement of our version of this Lemma and proof. The relationship yields the following corollary, with proof given in Supplementary Material A.

**Corollary 1** *(sNB of risk-based treatment policies and calibration of S at R ). Let S be a risk model for binary outcome Y that is increasing with event rate. Suppose S is used to select individuals for an intervention based on S>R , where R is a prespecified risk threshold that represents the benefits and harms of the intervention. Then S has maximum sNB among all recalibrated versions of S if and only if S is calibrated at R .*

### Graphically Assessing Potential Net Benefit under Recalibration

Before presenting our methods, we introduce a graphical tool to help researchers assess the potential for recalibration to improve the clinical utility of a risk model. Recalibration preserves the rank order of risk scores, meaning that under recalibration, some subset of individuals with similar predicted risks will move from below the risk threshold to above the risk threshold, or vice versa. Given fixed $\frac{C}{B}=\frac{R}{1-R}$ , for every $\vec{\alpha }$ that results in a new value of sNB , there is an equivalent cutpoint, r , that produces the same sNB when paired with the original risk score S . Using this relationship, varying the cutpoint between 0 and 1 for the original risk score S and harm-benefit ratio $\frac{R}{1-R}$ yields all values of sNB that can be achieved by recalibrating the risk model. We propose that investigators assess this space to understand the potential for recalibration to improve net benefit. Specifically, we propose that investigators plot estimates of

(2) $$\mathrm{sN}B_{r}=\mathrm{TP}R_{r}-\frac{R}{1-R}\frac{1-\pi }{\pi }\mathrm{FP}R_{r}$$

on the vertical axis against r∈[0,1] on the horizontal axis. We emphasize that R is constant in this expression of sNB because it represents benefits and harms. We note that when the cutpoint r equals the risk threshold R , equation (2) is the standardized net benefit of the risk model. In addition, when evaluated at the cutpoint r that maximizes $\mathrm{sN}B_{r}$ , equation (2) is the relative utility evaluated at risk threshold R .^27^

**Figure 1.**
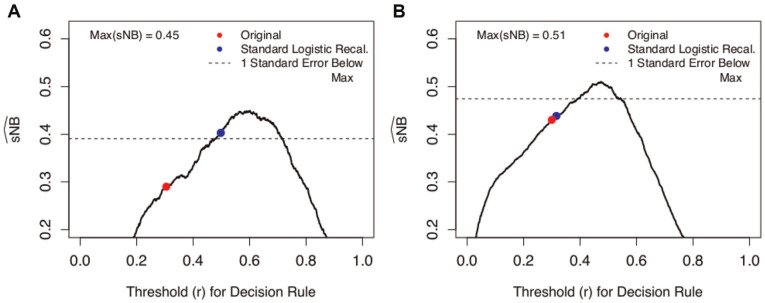
Potential sNB under recalibration. The dotted line shows 1 standard error below the estimated maximum possible sNB . In both (A) and (B), the estimated sNB for the original risk model is more than 1 standard error lower than the estimated maximum possible sNB , indicating that a recalibrated risk score could yield higher net benefit. In (A), the estimated sNB for the risk model after standard logistic recalibration is near the maximum value. Alternative methods of recalibration may not be worth pursuing in this setting. In (B), the recalibrated risk model produced by standard logistic recalibration yields estimated sNB more than 1 standard error lower than the estimated maximum possible sNB , suggesting that alternative recalibration methods may be useful.

Figure 1 shows 2 examples. In Figure 1, the horizontal axis gives all possible cutpoints, and the vertical axis gives $\hat{\mathrm{sNB}}$ for cutpoint r and fixed harm-benefit ratio $\frac{R}{1-R}$ . The maximum of the curve estimates the maximum sNB that can be achieved via recalibration of the risk model. The estimated sNB of the original risk score and the recalibrated risk score under standard logistic recalibration are noted on the curves, and these can be compared with the maximum. If the estimated sNB of the original risk score is far below the maximum of the curve, then there are potentially recalibration parameters $\left(\alpha_{0},\alpha_{1})\neq (0,1\right)$ that can increase the clinical utility of the risk model. Similarly, if standard logistic recalibration does not produce a risk model near the maximum, then alternative methods of recalibration may produce superior results. The graphical tool also provides researchers a sense of how much loss in sNB occurs due to miscalibration. From corollary 1, the maximum of this curve estimates the sNB of a risk score if calibrated at R . The vertical distance between the maximum of the curve and the observed sNB of the risk score estimates the loss in sNB from miscalibration at R .

In light of sampling variability, it may be unclear whether a risk model is “close” to the maximum. Following Friedman et al.,^29^ we suggest a “1-standard error” rule to decide if the estimated sNB of a risk model is near the maximum. Each plot in Figure 1 includes a dotted horizontal line 1 standard error below the maximum. (The standard error for the maximum of the curve is derived via the delta method; see Supplementary Material A.)

In both Figures 1A and 1B, the original risk model has notably lower $\hat{\mathrm{sNB}}$ than the maximum possible value. In Figure 1A, the estimated sNB of the risk model is close to the maximum possible value after recalibration via standard logistic recalibration. In contrast, standard logistic recalibration makes little difference in Figure 1B. Alternative methods of recalibration, such as those we propose, may be worthwhile to pursue in situations such as in Figure 1B.

### Weighted Logistic Recalibration for Improved Clinical Utility

We propose a weighted variant of Cox’s logistic recalibration to prioritize calibration near the risk threshold, which corollary 1 implies should maximize sNB . The weighted recalibration intercept $\alpha_{0}^{*}$ and slope $\alpha_{1}^{*}$ are estimated by maximizing the weighted likelihood

(3) $$L(\alpha_{0}^{*},\alpha_{1}^{*}|Y_{i},Z_{i})=\overset{n}{\underset{i=1}{\Pi }}p(Z_{i}{)}^{w_{i}Y_{i}}(1-p(Z_{i}{)}^{w_{i}(1-Y_{i})},$$

where $p(Z_{i})=\frac{1}{1+e^{-(\alpha_{0}^{*}+\alpha_{1}^{*}Z_{i})}}$ . We propose the weight function

(4) $$w_{i}=\left\{ \begin{array}{cc} \exp\left(-\frac{{\left(o(S_{i})-R\right)}^{2}}{\lambda }\right) & :o(S_{i})\in [R_{l},R_{u}]\\ \delta & :o(S_{i})\notin [R_{l},R_{u}] \end{array},\right.$$

where $o(S_{i})$ is a smoothed observed event rate, obtained via LOESS regression of $Y_{i}$ on the risk scores $S_{i}$ . Notation reflects the dependence of observed event rates on the risk model $S_{i}$ . $o(S_{i})$ are presented on the vertical axis of the calibration plot. λ and δ are tuning parameters and control the degree of differential weighting of observations. As λ increases, all weights tend to 1, and the weighted recalibration method approaches standard logistic recalibration. The parameter δ prescribes how much weight is assigned to observations outside a critical risk interval $\left[R_{l},R_{u}\right]$ , where clinicians may be additionally concerned about good calibration. δ is bounded below by 0 and bounded above by the infimum of the weights within the interval.

**Figure 2.**
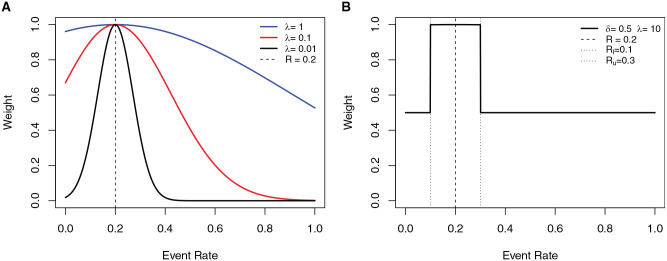
Example of weight functions used in the weighting scheme. The horizontal axis shows the event rate, and the vertical axis gives the weight. (A) Exponential decay weight with $\left[R_{l},R_{u}]\equiv [0,1\right]$ (and therefore no need to specify δ ) and λ=1,0.1, and 0.01 . This weight may be appropriate when good calibration at the risk threshold is of primary interest. (B) Weight function approximating a step function with λ=10 , δ=0.5 , and specified risk interval [10%,30%] around R=20% . This weighting scheme may be appropriate when good calibration over an interval of risk is of interest in addition to good calibration at R .

The weight function (4) encompasses 2 useful forms. The first has the form of an exponential decay weight (Figure 2A). Under this weighting scheme, observations with event rates at or near the risk threshold have the largest contribution to the likelihood, which decays exponentially moving away from R . The second form (Figure 2B) approximates a step function and is useful to prioritize calibration over a range of risks instead of a single risk threshold. In the ASCVD example, additional guidelines and current practices in cardiology indicate that 5% to 10% is an interval of critical risks that may affect clinical decisions. For settings in which good calibration is important for the interval $\left[R_{l},R_{u}\right]$ , λ can be set to a large value so that weights within the interval are all close to 1 (e.g., λ≥10 ), and only specification of δ is needed. For settings in which good calibration at the risk threshold R is most important, the exponential decay form can be used, and only specification of λ is needed. Supplementary Material B provides guidance for obtaining weights, including tuning parameter selection using a cross-validation procedure.

These weighting schemes down-weight, to a greater or lesser degree, observations with smoothed event rate ( $o(S_{i})$ ) away from the risk threshold. In a sense, we use less data to achieve a more targeted calibration, and therefore, there is a tradeoff between the improved calibration at or near the risk threshold (and therefore also sNB ) and the precision of results (more variability in ${\hat{\alpha }}_{0}$ and ${\hat{\alpha }}_{1}$ ). When using this method, we recommend reporting the effective sample proportion. Since all weights are between 0 and 1, the effective sample proportion can be calculated as the average weight, $\mathrm{Eff}=\frac{1}{n}\sum_{i=1}^{n}w_{i}.$ In standard logistic recalibration, all $w_{i}\equiv 1$ , and the effective sample proportion is 1.

### Constrained Logistic Recalibration

In our second approach to recalibration, we propose estimating the recalibration intercept and slope by maximizing the logistic likelihood over a restricted parameter space. The restricted space only includes recalibration parameters $\alpha_{0}$ and $\alpha_{1}$ that produce a recalibrated risk model with high sNB . The concepts in the “Graphically Assessing Potential Net Benefit under Recalibration” section make this possible, because we are able to estimate the maximum possible sNB among all possible relcalibrations of S .

Given a risk score S , risk threshold R , and recalibration parameters $\alpha_{0}$ and $\alpha_{1}$ , the plug-in estimator of the standardized net benefit of the recalibrated risk model is

(5) $$\hat{\mathrm{sNB}}(\alpha_{0},\alpha_{1})=\frac{1}{n_{Y}}\sum_{i=1}^{n_{Y}}1\left[\frac{e^{\alpha_{0}+\alpha_{1}Z_{i}}}{1+e^{\alpha_{0}+\alpha_{1}Z_{i}}}\;>\;R\right]-\frac{1-\hat{\pi }}{\hat{\pi }}\frac{R}{1-R}\frac{1}{n_{\bar{Y}}}\sum_{i=1}^{n_{\bar{Y}}}1\left[\frac{e^{\alpha_{0}+\alpha_{1}Z_{i}}}{1+e^{\alpha_{0}+\alpha_{1}Z_{i}i}}\;>\;R\right],$$

where $n_{Y}$ and $n_{\bar{Y}}$ are the number of cases and controls, respectively, in the sample of data available for recalibration. We propose estimating recalibration parameters $\vec{\alpha }=(\alpha_{0},\alpha_{1})$ via the following constrained maximization problem.

(6) $$\begin{array}{c} (\alpha_{0}^{*},\alpha_{1}^{*})=\underset{(\alpha_{0},\alpha_{1})\in R\times R^{+}}{\arg\max}\frac{1}{n}\sum_{i=1}^{n}\left[Y_{i}(\alpha_{0}+\alpha_{1}Z_{i})-\log\left(1+e^{\alpha_{0}+\alpha_{1}Z_{i}}\right)\right]\\ \;\;\;\mathrm{subject}\;\mathrm{to}\;\;\hat{\mathrm{sNB}}(\alpha_{0},\alpha_{1})\geq {\hat{\mathrm{sNB}}}_{\max}-\hat{\sigma }({\hat{\mathrm{sNB}}}_{\max}), \end{array}$$

where ${\hat{\mathrm{sNB}}}_{\max}$ is the estimated maximum achievable sNB among all risk scores of the form

$$S_{i}^{*}=\frac{e^{\alpha_{0}+\alpha_{1}Z_{i}}}{1+e^{\alpha_{0}+\alpha_{1}Z_{i}}}.$$

That is, we propose estimating $\vec{\alpha }$ by maximizing the likelihood of the logistic model over a constrained parameter space. For fixed harm-benefit ratio $\frac{R}{1-R}$ , ${\hat{\mathrm{sNB}}}_{\max}$ is found by varying decision threshold r (see the “Graphically Assessing Potential Net Benefit under Recalibration” section). That is, we solve the 1-dimensional optimization problem

$${\hat{\mathrm{sNB}}}_{\max}(S)=\underset{r\in [0,1]}{\max}\left\{{\hat{\mathrm{TPR}}}_{r}(S)-\frac{1-\hat{\pi }}{\hat{\pi }}\frac{R}{1-R}{\hat{\mathrm{FPR}}}_{r}(S)\right\}.$$

Acknowledging that there is uncertainty in ${\hat{\mathrm{sNB}}}_{\max}$ , we use a 1-standard-error type of rule in the inequality constraint. Such rules are often used when tuning penalized regression methods.^29^ The constrained parameter space includes all $\vec{\alpha }$ that produce a risk model with $\hat{\mathrm{sNB}}$ within 1 standard error of ${\hat{\mathrm{sNB}}}_{\max}$ . Supplementary Material A provides an estimate of this standard error.

The constrained logistic recalibration solution differs from the standard logistic recalibration solution when the latter is outside the constrained parameter space. These are exactly the instances in which there is evidence that standard logistic recalibration is inadequate in terms of the clinical utility of the recalibrated risk model. In situations lacking such evidence, the constrained and standard logistic recalibration solutions will be the same.

## Results

### Simulation Results

In this section, we compare weighted and constrained logistic recalibration to standard logistic recalibration using simulated data. We present 4 different simulation examples representing different types of miscalibration. For all examples, we use the risk threshold R=0.3 . For the weighted approach, an exponential and step weight function are used, with risk interval $\left[R_{l},R_{u}]=[0.25,0.35\right]$ . For brevity, results for the step function appear in Supplementary Material C. Tuning parameters are selected using 25 replications of 5-fold cross-validation.

Recalibration parameters are estimated from data sets of size 500, 1000, 5000, and 10,000. We use a large independent validation data set of size $10^{6}$ to evaluate the true (rather than estimated) risk model performance before and after recalibration. Table 6 in Supplementary Material C summarizes results for each example with 500 repeated simulations.

We simulate the data as follows. First, true risks ( $p_{i}$ ) are generated from a mixture Beta distribution, comprised of 3 subdistributions. The subdistributions are defined by the tendency to have low, medium, or high true risks. Beta hyperparameters and mixing proportions vary by example. Outcomes $Y_{i}$ are generated from a $\mathrm{Bern}(p_{i})$ distribution. The overall event rate is $E[Y_{i}]=E[E[Y_{i}|p_{i}]]=E[p_{i}]=\sum_{m=1}^{3}b_{3}\frac{\alpha_{3}}{\alpha_{3}+\beta_{3}}$ , where $\alpha_{m}$ and $\beta_{m}$ are the Beta hyperparameters for 3 different subpopulations, and $b_{m}$ is the mixing proportion for subpopulation m . Finally, we induce miscalibration by applying a piecewise polynomial function to the true risk model. We vary the type of miscalibration to capture different scenarios. Full details for each scenario are provided in Supplementary Material C.

**Figure 3:**
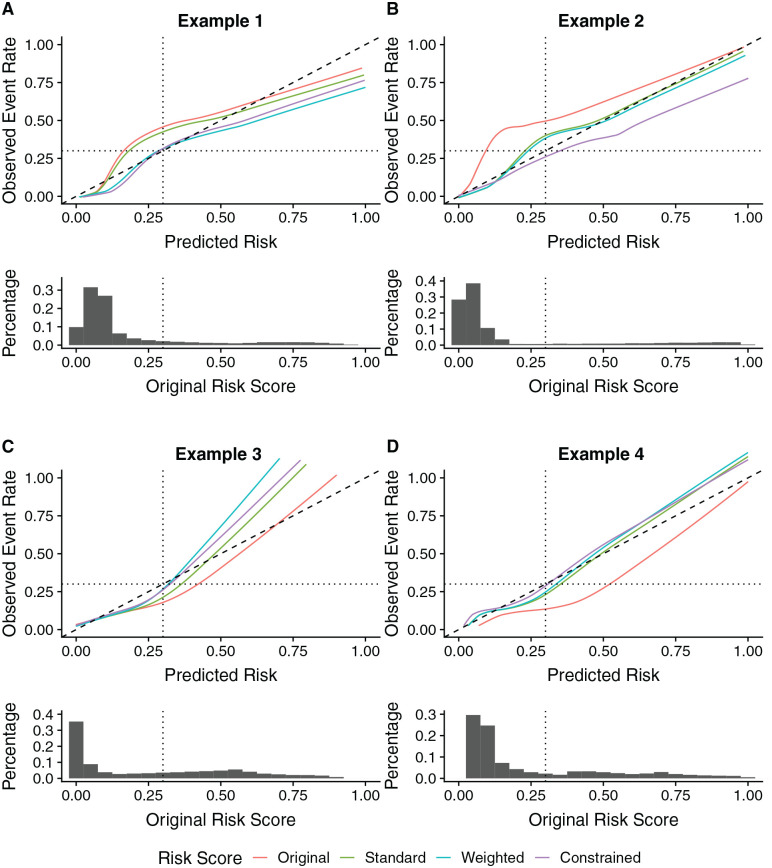
Calibration curves and the distributions of risk scores for examples 1 to 4 and sample size N=5000 . Calibration curves for the original, standard recalibrated, weighted recalibrated, and constrained recalibrated risk models are shown for each example. The histogram shows the distribution of risk scores before any recalibration. Dotted lines indicate the clinically important risk threshold, R=0.3 . The dashed is the identity line.

We present 4 types of miscalibration: underestimation of risk scores near the risk threshold and overestimation elsewhere (example 1); underestimation of risks for all risk scores (example 2); overestimation of risk scores near the risk threshold and underestimation far from the risk threshold (example 3); and overestimation of risks for all risk scores (example 4). Table 1 shows the sNB of the original and recalibrated risk models for all examples and sample sizes. Figure 3 shows calibration curves for all examples and sample size of N=5000 . Calibration plots for other sample sizes and additional simulation results are in Supplementary Material C.

**Table 1:** Summary of Simulation Results^a^

Sample Size	Original	Standard	Weighted	Constrained
Example 1				
500	0.430	0.446	0.446	0.458
1000	0.430	0.445	0.445	0.481
5000	0.430	0.455	0.503	0.504
10,000	0.430	0.457	0.499	0.501
Example 2				
500	0.475	0.510	0.513	0.524
1000	0.475	0.491	0.491	0.491
5000	0.475	0.511	0.523	0.524
10,000	0.475	0.511	0.521	0.525
Example 3				
500	0.440	0.459	0.461	0.475
1000	0.440	0.440	0.457	0.476
5000	0.440	0.458	0.484	0.488
10,000	0.440	0.449	0.502	0.503
Example 4				
500	0.282	0.417	0.418	0.417
1000	0.282	0.417	0.417	0.417
5000	0.282	0.413	0.421	0.435
10,000	0.282	0.422	0.430	0.431

a sNB of original risk score, and after standard, weighted, and constrained recalibration. All sNB calculations are obtained from a large set of independent data ( $N=10^{6}$ ) to show the true performance of each risk model.

In example 1, the original risk model underestimates risk at the risk threshold. The calibration curves in Figure 3A show good calibration at the risk threshold under the weighted and constrained approaches. In contrast, after standard recalibration, risks continue to be underestimated at the risk threshold. Weighted and constrained logistic recalibration increase sNB by 0.042 and 0.044 compared with standard logistic recalibration. However, gains are smaller for smaller sample sizes, particularly for the weighted approach. The smaller gains in sNB under the weighted approach can be attributed to the tuning parameter selected via the cross-validation procedure. When sample size is inadequate to support targeted recalibration, the weighted approach is designed to approximate standard logistic recalibration via the tuning parameter selection using the proposed cross-validation approach.

Next, we consider an example in which risks are underestimated across all predicted risks. The calibration curves shown in Figure 3B show slight improvement in calibration at the risk threshold for the weighted approach compared with standard logistic recalibration when N=5000 . Both weighted and constrained logistic recalibration yield a recalibrated risk model with larger sNB compared with standard logistic recalibration for all sample sizes except n=1000 . Weighted recalibration and constrained logistic recalibration produce similar sNB , with slightly higher sNB for the constrained approach. For the smallest sample size, n=500 , the constrained logistic recalibration approach has over 0.01 higher sNB compared with standard recalibration, while the weighted approach offers smaller improvement.

In this example, when the sample size is small, there are too little data near the risk threshold to support the weighted approach. Therefore, weighted recalibration approaches standard logistic recalibration. Similarly, when N=500 , the constrained logistic recalibration is the same as standard logistic recalibration because there is relatively large uncertainty in ${\hat{\mathrm{sNB}}}_{\max}$ , and the constraint space includes the standard logistic recalibration solution.

In example 3 (Figure 3C), risks are overestimated at the risk threshold and underestimated for very high and low predicted risks. Both the weighted and constrained recalibration methods produce a recalibrated risk model with higher sNB than standard logistic recalibration. As the sample size decreases, the sNB for weighted recalibration is similar to that for standard logistic recalibration, while the constrained recalibration approach has sustained increases in sNB compared with standard logistic recalibration. Weighted and constrained logistic recalibration sacrifice calibration away from the risk threshold to achieve better calibration near the risk threshold. These methods were designed to make this tradeoff, since miscalibration away from the risk threshold does not affect clinical decisions.^30^

Finally, in example 4, standard, weighted, and constrained logistic recalibration all have similar sNB when recalibration parameters are estimated in smaller data sets. For larger sample sizes, the sNB of the constrained approach is larger than all other methods, while the weighted method still offers higher sNB than standard logistic recalibration. For sample sizes N=500 and N=1000 , the estimated recalibration parameters under the weighted approach closely approximate those from standard recalibration (Table 5 in Supplementary Material C).

### Recalibration of the ACC-AHA-ASCVD Risk Model

**Figure 4:**
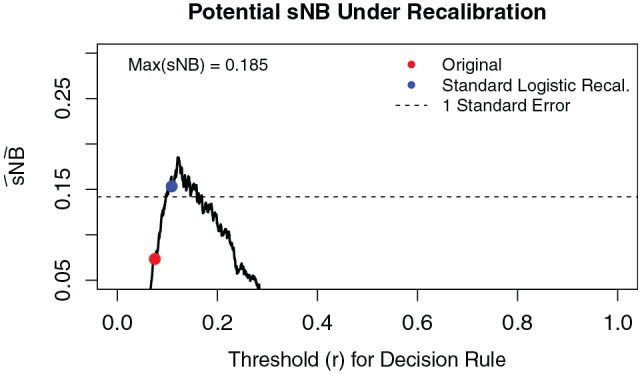
Potential gains in sNB under recalibration of the American College of Cardiology (ACC)–American Heart Association (AHA)–atherosclerotic cardiovascular disease (ASCVD) model for all Multi-Ethnic Study of Atherosclerosis participants eligible for risk score application (*N* = 4830). The plot indicates the potential for recalibration to achieve higher clinical utility than the original risk model since its estimated sNB is more than 1 standard error lower than the estimated maximum sNB . Standard logistic recalibration produces a risk model with near maximum $\hat{\mathrm{sNB}}$ , so results do not support pursuing specialized methods of recalibration.

MESA is a large, prospective, nationwide, multiethnic cohort study of cardiovascular disease (CVD) in men and women free of CVD at enrollment.^31^ Demographic and clinical data were collected at baseline, and participants were monitored for more than 10 y for cardiovascular clinical events. Recalibrating the ACC-AHA-ASCVD risk model using the MESA cohort and prioritizing good calibration at the treatment threshold of 7.5% could improve the clinical utility of the risk tool for the population. Figure 4 shows the estimated potential sNB of the ACC-AHA-ASCVD risk model. After standard logistic recalibration, the estimated sNB is near the maximum, suggesting that alternative recalibration methods may not be worthwhile.

**Figure 5:**
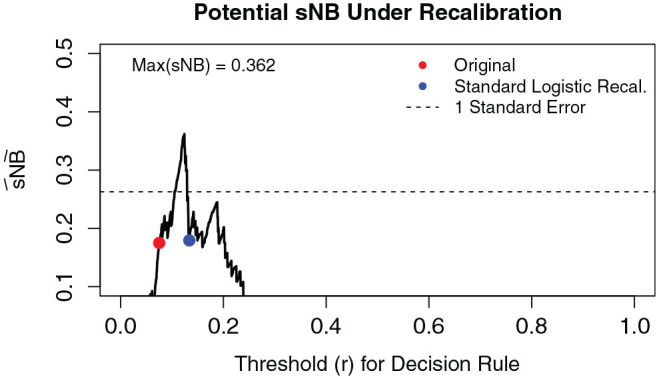
Potential gains in sNB under recalibration of the American College of Cardiology (ACC)–American Heart Association (AHA)–atherosclerotic cardiovascular disease (ASCVD) risk model for Black males eligible for risk score application (*N* = 538). The plot indicates the potential to achieve higher clinical utility than the original risk model or the risk model after standard logistic recalibration since the estimated sNB of those risk models is more than 1 standard error lower than the estimated maximum sNB .

MESA is an ethnically diverse cohort, and there is interest in evaluating and correcting miscalibration of the ACC-AHA-ASCVD risk score within different subgroups defined by sex and/or ethnicity.^2^ Applying the graphical tool to different subgroups in MESA, we found potential for improvement for the Black male cohort (Figure 5). The 10-y event rate of CVD in Black men (within age range and low-density lipoprotein range, and diabetes free, N=538 ) was 7.1%. The average estimated 10-y risk of CVD from the ACC-AHA-ASCVD risk score was 12.5%, indicating overestimation of risks.

**Figure 6:**
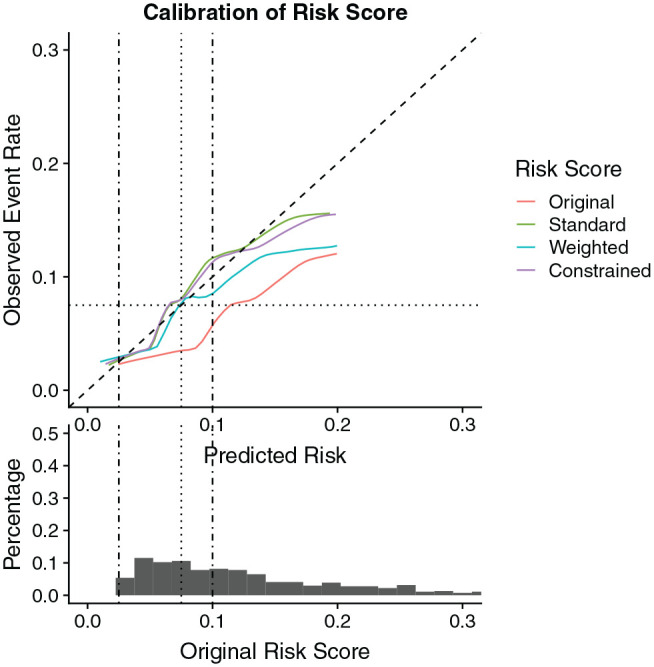
Calibration curves for the original risk score as well as standard, weighted, and constrained recalibrated risk score in the Multi-Ethnic Study of Atherosclerosis Black, male cohort.

We applied standard, weighted, and constrained logistic recalibration to the ACC-AHA-ASCVD risk score in the Black, male MESA cohort. Table 2 shows the estimated recalibration parameters $\hat{\alpha }$ , standardized net benefit (and its components), event rate in the risk interval, and proportion treated. We used bootstrap methods to correct for optimistic bias in estimating sNB .^8^ The estimated maximum achievable sNB under recalibration for this sample was 0.362, with estimated standard error 0.102. Therefore, the lower bound used to to define the constrained parameter space was $\hat{\mathrm{sNB}}(\vec{\alpha })=0.260$ . Both weighted and constrained recalibration offered improvements in sNB over standard recalibration. Figure 6 shows similar calibration between the three methods at the risk threshold.

**Table 2:** Comparison of Recalibration Methods in the MESA Black, Male Cohort for RAW = 0.01^a^

Measure	Original	Standard	Weighted	Constrained
( ${\hat{\alpha }}_{0}$ , ${\hat{\alpha }}_{1}$ )	—	(–0.911, 0.856)	(0.088, 1.271)	(–0.699, 0.960)
Effective Sample Proportion %	—	100	70	100
$\hat{\mathrm{sNB}}$ (95% CI)	0.175 (–0.082, 0.432)	0.179 (–0.068, 0.426)	0.304 (0.023, 0.586)	0.274 (–0.018, 0.473)
Optimism corrected $\hat{\mathrm{sNB}}$ (95% CI)	—	0.165 (–0.082, 0.411)	0.295 (0.013, 0.577)	0.211 (–0.034 0.457)
Optimism corrected $\hat{\mathrm{TPR}}$	0.868	0.550	0.759	0.582
Optimism corrected $\hat{\mathrm{FPR}}$	0.650	0.350	0.430	0.355

a For weighted recalibration, an indicator weight is used where a constant weight is applied to observations within the clinically defined risk interval [2.5%, 10%], and a smaller constant weight is applied to observations outside that interval. Optimism-bias correction for $\hat{\mathrm{sNB}}$ and confidence intervals estimated via bootstrap method with 500 replications.

We acknowledge wide confidence intervals in these results. The small sample size and resulting uncertainty make it difficult to draw definitive conclusions about improved clinical utility. However, despite the small sample size, both the graphical device and optimism-corrected estimates of sNB suggest the proposed methods are advantageous.

## Discussion

We presented methods for risk model recalibration that aim to optimize a risk model’s clinical utility for making risk-based decisions. Box 1 compares the 2 proposed methods, which are both generalizations of standard logistic recalibration. Moreover, both methods can be expected to approximate or reproduce standard logistic recalibration when it produces good calibration at the critical risk threshold. We consider this feature a strength of these approaches.

**Box 1:** Comparison of Proposed Recalibration Methods

*Weighted logistic recalibration*
• Aims to improve recalibration at the risk threshold, with improved sNB as result
• Flexible weight function allows researchers to specify either a single risk or an interval where good calibration should be prioritized
• Down-weighting reduces effective sample size
• Requires tuning parameters
*Constrained logistic recalibration*
• Aims to maximize sNB , with improved calibration at the risk threshold as a consequence
• If variance of ${\hat{\mathrm{sNB}}}_{\max}$ is large, the constrained parameter space will also be large, and the recalibration will be identical to standard logistic recalibration

We additionally proposed a graphical device to help researchers assess the potential for recalibration to improve the clinical utility of a risk model. For a predefined risk model, we also provided methods to estimate its maximum possible net benefit and its variance. These results enable researchers to evaluate whether specialized methods of recalibration, such as the 2 we propose, are likely to be advantageous. Both methods and the graphical tools are in the R package ClinicalUtilityRecal.^32^

As discussed, this work assumes all conditions required for net benefit metrics to be meaningful. We also emphasize that we do not think recalibration should be an automatic response to observing miscalibration. Miscalibration can indicate issues, such as measurement or population heterogeneity, that might be resolved in other ways. When possible, identifying the source of miscalibration can provide researchers with a better understanding of avenues for correction, as well as indications of complex changes in populations. Moreover, if there are adequate data to develop a new risk model, refitting may be preferred over recalibration. Other work has compared standard logistic recalibration to refitting methods.^33–35^ However, even when refitting is possible, investigators might prefer recalibration to maintain a connection with the original model. In this article, we presume a context in which investigators have decided that recalibration is their best course of action.

Standard logistic recalibration is a parsimonious method to address miscalibration. In settings where the miscalibration pattern at the risk threshold is similar to the pattern for the bulk of the data (e.g., systematic under- or overestimation) or settings where there is under- or overfitting, standard logistic recalibration may adequately improve calibration at the risk threshold. In settings where standard logistic recalibration does not adequately correct miscalibration at the risk threshold, alternative recalibration methods are useful to ensure risk-guided clinical decisions are made appropriately. However, it may be unappealing to use methods that increase the number of recalibration parameters estimated, particularly if this leads to overfitting. Our methods leverage the parsimony of standard logistic recalibration while allowing researchers to focus on the regions where good calibration matters most. Furthermore, we note that our methods could naturally be applied with other families of recalibration functions, such as the 3-parameter family proposed by Kull et al.^36^ The methods we propose are not intrinsically tied to the logistic recalibration family.

Statistical software may return elements of statistical inference (standard errors, confidence intervals, *P* values) when estimating the recalibration intercept and slope. These elements might be useful when the model is fit to detect miscalibration, but we do not find them to be useful for the actual process of recalibration. Instead, there are 2 instances in which elements of statistical inference play a key role in our proposed methods. First, we propose a 1-standard-error rule for assessing the potential for recalibration to improve clinical utility using our proposed graphical device. Second, we suggest that investigators use a 1-standard-error rule when implementing constrained logistic recalibration.

Weighted logistic recalibration requires tuning parameters to specify the weighting scheme. We envision using the weighting scheme in 1 of 2 special forms, each requiring a single tuning parameter. The computational burden of cross-validation is a disadvantage of the weighted method. When there are few events, heavy down-weighting may be undesirable. In these instances, the cross-validation procedure paired with a 1-standard-error rule will indicate that the data do not support the weighted approach, and weighted logistic recalibration will approximate standard logistic recalibration. In general, we recommend reporting the effective sample proportion to gauge the impact of weighting.

As risk prediction becomes more ubiquitous with the increase in both data availability and more sophisticated prediction methods, opportunities to observe miscalibration are also more common. A recent article describes and classifies reasons for “data set drift” and implications for the performance of artificial intelligence systems.^5^ A risk model’s miscalibration has been called “clinically harmful” if it reduces the net benefit of using the risk model below that of the uniform treatment policies (treat all and treat none).^25^ However, Kerr et al.^37^ give an example in which the net benefit of a miscalibrated risk model is higher than both uniform treatment policies, but addressing the miscalibration could substantially improve the model’s net benefit to the relevant population. This is a situation in which we consider miscalibration to be clinically harmful. It is important to assess calibration even if a risk model outperforms treat-all and treat-none rules. Investigators should consider recalibrating a risk model whenever there is evidence that its clinical utility could be meaningfully improved.

## Supplemental Material

sj-pdf-1-mdm-10.1177_0272989X211044697 – Supplemental material for Recalibration Methods for Improved Clinical Utility of Risk Scores Click here for additional data file. Supplemental material, sj-pdf-1-mdm-10.1177_0272989X211044697 for Recalibration Methods for Improved Clinical Utility of Risk Scores by Anu Mishra, Robyn L. McClelland, Lurdes Y. T. Inoue and Kathleen F. Kerr in Medical Decision Making

## References

[1] Goff DC Jr Lloyd-Jones DM Bennett G , et al. 2013 ACC/AHA guideline on the assessment of cardiovascular risk. Circulation. 2014;129(25 suppl 2):S49–73. 10.1161/01.cir.0000437741.48606.98 24222018

[2] DeFilippis AP Young R Carrubba CJ , et al. An analysis of calibration and discrimination among multiple cardiovascular risk scores in a modern multiethnic cohort. Ann Intern Med. 2015;162(4):266–75. 10.7326/M14-1281 PMC4414494 25686167

[3] Ridker PM Cook NR . Statins: new American guidelines for prevention of cardiovascular disease. Lancet. 2013;382(9907):1762–5. 10.1016/S0140-6736(13)62388-0 24268611

[4] Davis SE Lasko TA Chen G Siew ED Matheny ME . Calibration drift in regression and machine learning models for acute kidney injury. J Am Med Inform Assoc. 2017;24(6):1052–61. 10.1093/jamia/ocx030 PMC6080675 28379439

[5] Finlayson SG Subbaswamy A Singh K , et al. The clinician and dataset shift in artificial intelligence. N Engl J Med. 2021;385:283–6. 10.1056/NEJMc2104626 PMC8665481 34260843

[6] Van Calster B Nieboer D Vergouwe Y De Cock B Pencina MJ Steyerberg EW . A calibration hierarchy for risk models was defined: from utopia to empirical data. J Clin Epidemiol. 2016;74:167–76. 10.1016/j.jclinepi.2015.12.005 26772608

[7] Hosmer DW Jr Lemeshow S Sturdivant RX . Applied Logistic Regression. Vol. 398. New York: John Wiley & Sons; 2013.

[8] Harrell F . Regression Modeling Strategies: With Applications to Linear Models, Logistic and Ordinal Regression, and Survival Analysis. New York: Springer; 2015.

[9] Cox DR . Two further applications of a model for binary regression. Biometrika. 1958;45(3/4):562–5.

[10] Dalton JE . Flexible recalibration of binary clinical prediction models. Stat Med. 2013;32(2):282–9. 10.1002/sim.5544 22847754

[11] Platt JC . Probabilistic outputs for support vector machines and comparisons to regularized likelihood methods. Adv Large Margin Classifiers. 1999;10(3):61–74.

[12] Zadrozny B Elkan C . Obtaining calibrated probability estimates from decision trees and naive Bayesian classifiers. In: ICML. Vol. 1. Citeseer; 2001. p 609–16.

[13] Zadrozny B Elkan C . Transforming classifier scores into accurate multiclass probability estimates. In: Proceedings of the 8th ACM SIGKDD International Conference on Knowledge Discovery and Data Mining; ACM; 2002. p 694–9.

[14] Niculescu-Mizil A Caruana R . Predicting good probabilities with supervised learning. In: Proceedings of the 22nd International Conference on Machine Learning; ACM; 2005. p 625–32.

[15] Jiang X Osl M Kim J Ohno-Machado L . Calibrating predictive model estimates to support personalized medicine. J Am Med Inform Assoc. 2011;19(2):263–74. 10.1136/amiajnl-2011-000291 PMC3277613 21984587

[16] Baker SG Cook NR Vickers A Kramer BS . Using relative utility curves to evaluate risk prediction. J R Stat Soc Ser A Stat Soc. 2009;172(4):729–48. 10.1111/j.1467-985X.2009.00592.x PMC2804257 20069131

[17] Vickers AJ Elkin EB . Decision curve analysis: a novel method for evaluating prediction models. Med Decis Making. 2006;26(6):565–74. 10.1177/0272989X06295361 PMC2577036 17099194

[18] Steyerberg EW Vickers AJ . Decision curve analysis: a discussion. Med Decis Making. 2008;28(1):146–9. 10.1177/0272989X07312725 PMC2577563 18263565

[19] Pepe M Janes H . Methods for Evaluating Prediction Performance of Biomarkers and Tests. New York: Springer; 2013.

[20] Kerr KF Brown MD Zhu K Janes H . Assessing the clinical impact of risk prediction models with decision curves: guidance for correct interpretation and appropriate use. J Clin Oncol. 2016;34(21):2534–40. 10.1200/JCO.2015.65.5654 PMC4962736 27247223

[21] Pauker SG Kassirer JP . The threshold approach to clinical decision making. N Engl J Med. 1980;302(20):1109–17. 10.1056/NEJM198005153022003 7366635

[22] Baker SG Kramer BS . Peirce, Youden, and receiver operating characteristic curves. Am Stat. 2007;61(4):343–6.

[23] Kerr KF Marsh TL Janes H . The importance of uncertainty and opt in vs. opt out: best practices for decision curve analysis. Med Decis Making. 2019;39(5):491–2. 10.1177/0272989X19849436 PMC6786944 31104561

[24] Kerr KF Brown MD Marsh TL Janes H . Assessing the clinical impact of risk models for opting out of treatment. Med Decis Making. 2019;39(2):86–90. 30649998 10.1177/0272989X18819479 PMC6374190

[25] Van Calster B Vickers AJ . Calibration of risk prediction models impact on decision-analytic performance. Med Decis Making. 2015;35(2):162–9. 10.1177/0272989X14547233 25155798

[26] Pepe MS Fan J Feng Z Gerds T Hilden J . The net reclassification index (NRI): a misleading measure of prediction improvement even with independent test data sets. Stat Biosci. 2015;7(2):282–95. 10.1007/s12561-014-9118-0 PMC4615606 26504496

[27] Baker SG Van Calster B Steyerberg EW . Evaluating a new marker for risk prediction using the test tradeoff: an update. Int J Biostat. 2012;8(1):1–37. 10.1515/1557-4679.1395 22499728

[28] Metz CE . Basic principles of ROC analysis. In: Seminars in Nuclear Medicine. Vol. 8. Amsterdam: Elsevier; 1978. p 283–98. 10.1016/s0001-2998(78)80014-2 112681

[29] Friedman J Hastie T Tibshirani R . The Elements of Statistical Learning. Vol. 1. Springer Series in Statistics. New York: Springer; 2001.

[30] Vickers AJ Van Calster B Steyerberg E . Decision curves, calibration, and subgroups. J Clin Oncol. 2017;35(4):472–3. 10.1200/JCO.2016.69.1576 28129527

[31] Bild DE Bluemke DA Burke GL Detrano R Diez Roux AV Folsom AR , et al. Multi-ethnic study of atherosclerosis: objectives and design. Am J Epidemiol. 2002;156(9):871–81. 10.1093/aje/kwf113 12397006

[32] Mishra A . ClinicalUtilityRecal: recalibration methods for improved clinical utility of risk scores; 2020. R package version 0.1.0. Available from: https://CRAN.R-project.org/package=ClinicalUtilityRecal 10.1177/0272989X211044697 PMC8977399 34605718

[33] Steyerberg EW Borsboom GJ van Houwelingen HC Eijkemans MJ Habbema JDF . Validation and updating of predictive logistic regression models: a study on sample size and shrinkage. Stat Med. 2004;23(16):2567–86. 10.1002/sim.1844 15287085

[34] Vergouwe Y Nieboer D Oostenbrink R Debray TP Murray GD Kattan MW , et al. A closed testing procedure to select an appropriate method for updating prediction models. Stat Med. 2017;36(28):4529–39. 10.1002/sim.7179 27891652

[35] Steyerberg EW Harrell FE Jr Borsboom GJ Eijkemans M Vergouwe Y Habbema JDF . Internal validation of predictive models: efficiency of some procedures for logistic regression analysis. J Clin Epidemiol. 2001;54(8):774–81. 10.1016/s0895-4356(01)00341-9 11470385

[36] Kull M Silva Filho T Flach P . Beta calibration: a well-founded and easily implemented improvement on logistic calibration for binary classifiers. In: Artificial Intelligence and Statistics; Proceedings of Machine Learning Research; 2017. p 623–31.

[37] Kerr KF Brown M Janes H . Reply to AJ Vickers et al. J Clin Oncol. 2016;35(4):473–5. 10.1200/JCO.2016.70.4288 28129522

