# Supplementary material for: Recalibration Methods for Improved Clinical Utility of Risk Scores
Source: Med Decis Making. 2021 Oct 4;42(4):500–12. doi: 10.1177/0272989X211044697 (PMC8977399; doi:10.1177/0272989X211044697)
Supplement: sj-pdf-1-mdm-10.1177_0272989X211044697 – Supplemental material for Recalibration Methods for Improved Clinical Utility of Risk Scores [file sj-pdf-1-mdm-10.1177_0272989X211044697.pdf]

Supplementary Material for Recalibration Methods for Improved Clinical Utility of Risk Scores

Anu Mishra, Robyn L. McClelland, Lurdes Y.T. Inoue, Kathleen F. Kerr

A Theoretical Statements and Proofs

The results in Lemma 1 and Corollary 1 assume concave ROC curve, which follows from the monotonicity of the risk models we consider in this article. We note that an alternative statement and proof of Lemma 1 by Baker et al. (2012)[1] (equation 6).

Lemma 1. *(risk model calibration and ROC slope.) Let S be a risk model monotonically increasing with event rate for binary outcome Y and let $r \in (0,1)$. Then*

$$P[Y=1|RS=r] = \frac{1}{1+ROCSLOPE^{-1}\frac{1-\pi}{\pi}}, \tag{1}$$

where ROCSLOPE is the slope of the ROC curve of risk score S at cutpoint r.

Proof. Let S be a risk score. Let $f_{S|Y=1}(rs)$ and $f_{S|Y=0}(rs)$ represent the densities of S for cases and controls, respectively. Let r_ϵ be an ϵ-width interval around cutpoint r, where $r \in (0,1)$. Here we relate the height of the calibration curve at r and the slope of the ROC curve for cutpoint r.

$$\begin{aligned}
P(Y=1|S=r) = \lim_{\epsilon\to 0} P(Y=1|S\in r_\epsilon) &= \lim_{\epsilon\to 0}\frac{P(S\in r_\epsilon|Y=1)P(Y=1)}{P(S\in r_\epsilon)}\\
&= \lim_{\epsilon\to 0}\frac{P(S\in r_\epsilon|Y=1)P(Y=1)}{P(S\in r_\epsilon|Y=1)P(Y=1)+P(S\in r_\epsilon|Y=0)P(S\in r_\epsilon|Y=0)P(Y=0)}\\
&= \lim_{\epsilon\to 0}\frac{1}{1+\frac{P(S\in r_\epsilon|Y=0)P(Y=0)}{P(S\in r_\epsilon|Y=1)P(Y=1)}}\\
&= \lim_{\epsilon\to 0}\frac{1}{1+\frac{f_{S|Y=0}(r_\epsilon)}{f_{S|Y=1}(r_\epsilon)}\frac{1-\pi}{\pi}}\\
&= \frac{1}{1+ROCSLOPE(r)^{-1}\frac{1-\pi}{\pi}},
\end{aligned}$$

where ROCSLOPE(r) is the slope of the ROC curve at the point corresponding to cutpoint r. The last equality uses an established relationship (Result 4.3 in Pepe (2003)[2]). □

Using the results of the above Lemma, we arrive at the following corollary. We note that this corollary also follows directly from optimization requirement shown in Baker et al. (2009).[3]

Corollary 1. *(sNB of risk-based treatment policies and calibration of S at R.) Let S be a risk score for binary outcome Y, that is monotonically increasing with event rate. Suppose S is used to select individuals for an intervention based on $S > R$, where R is a pre-specified risk threshold that represents the benefits and harms of the intervention. Then S has maximum sNB among all recalibrated versions of S if and only if S is calibrated at R.*

Proof. For a risk score, S with differentiable and concave ROC curve, the cutpoint r that maximizes sNB has

$$ROCSLOPE(r) = \frac{1-\pi}{\pi}\frac{C}{B}.$$

Let R be the risk threshold corresponding to the harms and benefits of an intervention, i.e., $\frac{R}{1-R} = \frac{C}{B}$. Risk score S is calibrated at R if and only if R maximizes sNB among all possible cutpoints r. Suppose S is calibrated at R, i.e., $P(Y = 1|RS) = R$. Then

$$R = \frac{1}{1 + ROCSLOPE(R)^{-1}\frac{1-\pi}{\pi}}$$
$$\Rightarrow ROCSLOPE(R) = \frac{1-\pi}{\pi}\frac{C}{B}.$$

Suppose R maximizes sNB among all possible cutpoints for risk score S. Then $ROCSLOPE(R)$ is

$$ROCSLOPE(R) = \frac{1-\pi}{\pi}\frac{C}{B}$$
$$\Rightarrow P(Y = 1|RS = R) = \frac{1}{1 + \frac{\pi}{1-\pi}\frac{1-R}{R}\frac{1-\pi}{\pi}} = R,$$

and S is calibrated at R. □

Proposition. *(variance of $\widehat{sNB}(S)$ at cutpoint r.)*
Let S be a risk model predicting binary random variable Y. Assume that the harm to benefit ratio of the intervention associated with the outcome is $\frac{R}{1-R}$. For a random sample of n individuals from the relevant population, the estimated standardized net benefit of the policy that treats individuals with $S > r$ is

$$\widehat{sNB}\,(S) = \widehat{TPR}_r(S) - \frac{R}{1-R}\frac{\hat{P}(Y=0)}{\hat{P}(Y=1)}\widehat{FPR}_r(S) \tag{2}$$

Define the sample proportions:

$$\hat{p}_{11} = \frac{1}{n}\mathbf{1}[S \geq r, Y = 1] \qquad \hat{p}_{01} = \frac{1}{n}\mathbf{1}[S < r, Y = 1], \tag{3}$$
$$\hat{p}_{10} = \frac{1}{n}\mathbf{1}[S \geq r, Y = 0] \qquad \hat{p}_{00} = \frac{1}{n}\mathbf{1}[S < r, Y = 0]. \tag{4}$$

The estimator in (2) can be expressed as a function of $\vec{\hat{p}} = \{\hat{p}_{11}, \hat{p}_{10}, \hat{p}_{01}, \hat{p}_{00}\}$, when $\hat{p}_{11} + \hat{p}_{01} > 0$ and $\hat{p}_{10} + \hat{p}_{00} > 0$ (since we can never divide by 0),

$$\widehat{sNB}(\vec{\hat{p}}) = \frac{\hat{p}_{11}}{\hat{p}_{11} + \hat{p}_{01}} - \frac{C}{B}\left(\frac{\hat{p}_{10} + \hat{p}_{00}}{\hat{p}_{11} + \hat{p}_{01}}\right)\frac{\hat{p}_{10}}{\hat{p}_{10} + \hat{p}_{00}}.$$

Then, the variance of $\widehat{sNB}_t(\vec{\hat{p}})$ given harm to benefit ratio, $\frac{C}{B}$, is

$$V\left[\widehat{sNB}\left(\vec{\hat{p}}\right)\right] = \left(\frac{1}{p_{11} + p_{01}}\right)^2 \left[\left(\frac{R}{1-R}\right)^2 p_{10} + \frac{p_{11}\left(p_{01} + \frac{R}{1-R}p_{10}\right)^2}{(p_{11} + p_{01})^2} + \frac{p_{01}(\frac{C}{B}p_{10} - p_{11})^2}{(p_{11} + p_{01})^2}\right], \tag{5}$$

where

$$p_{11} = P(S \geq r, Y = 1) \qquad p_{01} = P(S < r, Y = 1), \tag{6}$$
$$p_{10} = P(S \geq r, Y = 0) \qquad p_{00} = P(S < r, Y = 0), \tag{7}$$

and $p_{11} + p_{01} > 0$ and $p_{10} + p_{00} > 0$.

Proof. For $i = 1, \dots, n$ define

$$N_{11} = \sum_{i=1}^{n} \mathbf{1}[S_i \geq r, Y_i = 1] \qquad N_{01} = \sum_{i=1}^{n} \mathbf{1}[S_i < r, Y_i = 1]$$
$$N_{10} = \sum_{i=1}^{n} \mathbf{1}[S_i \geq r, Y_i = 0] \qquad N_{00} = \sum_{i=1}^{n} \mathbf{1}[S_i < r, Y_i = 0]$$

with $N_{11} + N_{10} + N_{01} + N_{00} = n$. The counts N_{11}, N_{10}, N_{01}, and N_{00} follow a multinomial distribution with corresponding probabilities,

$$p_{11} = P(S \geq r, Y = 1) \qquad p_{01} = P(S < r, Y = 1), \tag{8}$$
$$p_{10} = P(S \geq r, Y = 0) \qquad p_{00} = P(S < r, Y = 0), \tag{9}$$

which have maximum likelihood estimator $\hat{p}_{lk} = \frac{N_{lk}}{n}$ for $l = 1, 2$ and $j = 1, 2$. By the multivariate central limit theorem

$$\sqrt{n} \begin{Bmatrix} \hat{p}_{11} - p_{11} \\ \hat{p}_{10} - p_{10} \\ \hat{p}_{01} - p_{01} \\ \hat{p}_{00} - p_{00} \end{Bmatrix} \xrightarrow{d} N\left(\vec{0}, \Sigma\right),$$

where

$$\Sigma = \begin{Bmatrix} p_{11}(1-p_{11}) & -p_{11}p_{10} & -p_{11}p_{01} & -p_{11}p_{00} \\ -p_{11}p_{10} & p_{10}(1-p_{10}) & -p_{10}p_{01} & -p_{10}p_{00} \\ -p_{11}p_{01} & -p_{10}p_{01} & p_{01}(1-p_{01}) & -p_{01}p_{00} \\ -p_{11}p_{00} & -p_{10}p_{00} & -p_{01}p_{00} & p_{00}(1-p_{00}) \end{Bmatrix}.$$

The estimand of (2) can be expressed as a function of $\vec{p} = \{p_{11}, p_{10}, p_{01}, p_{00}\}$,

$$\begin{aligned} sNB(\vec{p}) &= \frac{p_{11}}{p_{11} + p_{01}} - \frac{R}{1-R} \left(\frac{p_{10} + p_{00}}{p_{11} + p_{01}}\right) \frac{p_{10}}{p_{10} + p_{00}} && (10) \\ &= \frac{p_{11}}{p_{11} + p_{01}} - \frac{R}{1-R} \frac{p_{10}}{p_{11} + p_{01}} \\ &= \frac{1}{p_{11} + p_{01}} [p_{11} - \frac{R}{1-R} p_{10}]. && (11) \end{aligned}$$

The gradient of (11) is

$$\nabla sNB_t(\vec{p}) = \begin{Bmatrix} \frac{p_{01} + \frac{C}{B} p_{10}}{(p_{11} + p_{01})^2} \\ \frac{-\frac{R}{1-R}}{p_{11} + p_{01}} \\ \frac{-p_{11} + \frac{R}{1-R} p_{10}}{(p_{11} + p_{01})^2} \\ 0 \end{Bmatrix}$$

Then by the invariance property of the maximum likelihood estimator and invoking the δ-method,

$$\sqrt{n}\left(\widehat{sNB}(\vec{\hat{p}}) - sNB(\vec{p})\right) \xrightarrow{d} N(0, \Sigma_2),$$

where

$$\begin{aligned} \Sigma_2 &= \nabla sNB(\vec{p})^T \Sigma \nabla sNB(\vec{p}) \\ &= \left(\frac{1}{p_{11} + p_{01}}\right)^2 \left[\left(\frac{R}{1-R}\right)^2 p_{10} + \frac{p_{11}\left(p_{01} + \frac{R}{1-R} p_{10}\right)^2}{(p_{11} + p_{01})^2} + \frac{p_{01}(\frac{R}{1-R} p_{10} - p_{11})^2}{(p_{11} + p_{01})^2}\right]. \end{aligned}$$

□

B Calculating Weights for Weighted Logistic Recalibration

As described in section 2.3, the weight function used in weighted logistic recalibration has flexible forms. The choice of weight function depends on the context in which the risk score will be used and where good calibration of the risk score is most important. In the following section we provide more detail on construction of the weight functions and guidance for selecting associated tuning parameters, including a cross-validation procedure.

B.1 Weight components and weight shape

The weight function is comprised of five components: the risk threshold R, the smoothed observed event rate $o(RS_i)$, the critical risk interval $[R_l, R_u]$ containing R, and tuning parameters λ and δ. The smoothed observed event rate is obtained from a LOESS regression of Y_i on RS_i, and is used to identify observations with event rate close to R. The critical risk interval defines a range of risk scores around the risk threshold where calibration has heightened clinical importance. Finally, δ sets a constant weight outside the critical risk interval and λ sets the rate at which observations further from R are down-weighted.

As shown in Figure 2 in the main text, the weight function can take an exponential or step function form. Depending on the desired shape of the weight function some parameters may be considered ancillary. Under a fully exponential decay weight shape, as pictured in Figure 2(A) of the main text, recalibration is focused at the risk threshold. In this setting there may not be an already defined clinically relevant critical risk intervals, and one can set $[R_l, R_u] = [0, 1]$. Then, δ is not a relevant parameter for the weight function; only λ requires specification. Under the step function weight form, as pictured in Figure 2(B) of the main text, the critical risk interval and δ should be specified, and λ can be arbitrarily large (e.g $\lambda \geq 10$) to achieve a "flat" weight function within the critical risk interval. Variations between these weight forms can be easily achieved by varying λ, δ, or $[R_l, R_u]$.

Before selecting a tuning parameter, we recommend that researchers first select the general shape of the weight (exponential, step, or some intermediary). Specifying the shape will identify which tuning parameters are necessary to specify, and which tuning parameters are ancillary.

B.2 Tuning parameter selection and relative average weights (RAW)

Once the general shape is chosen the next step is to select tuning parameters. However, neither λ nor δ has a clinical interpretation, which contributes to the challenge of selecting these tuning parameters.

We define a more interpretable measure, *the relative average weight* (RAW), that can be used to elicit or select λ or δ. RAW compares the average weight of observations within $[R_l, R_u]$ to the average weight of observations outside the interval. For the step function weight form, the critical risk interval is already defined. For exponential decay weights, such as shown in Figure 2(A), $[R_l, R_u]$ is selected and used exclusively for defining RAW and is not otherwise used in the weighting scheme. We define the relative average weight

$$RAW = \frac{n_R}{n_{\bar{R}}} \frac{\sum_{i=1}^{n} w_i \mathbf{1}[o(S_i) \notin (R_l, R_u)]}{\sum_{i=1}^{n} w_i \mathbf{1}[o(S_i) \in (R_l, R_u)]}, \tag{12}$$

where n_R ($n_{\bar{R}}$) is the number of observations within (outside) the interval $[R_l, R_u]$.

A useful interpretation of RAW is a summary metric capturing how much down-weighting is applied. RAW is bounded by (0,1). A RAW value close to 1 implies that there is little differential weighting and λ or δ are large. RAW near 0 implies that the average weight for an observation outside the interval is much smaller than the average weight for observations within the interval. RAW close to 0 implies substantial down-weighting of observations far from the risk threshold and corresponds to a small value of λ or δ.

If there is a prior notion of how much observations outside the interval should be down-weighted, λ or δ can be identified by fixing one parameter (depending on desired weight shape) and numerically solving for the other using (12). Otherwise, a range of RAW values can be used to obtain a grid of λ (or δ for step weight function). We propose a cross-validation procedure to select the tuning parameter based on maximizing sNB.

B.3 Cross-validation for tuning parameter selection

In this section we present a repeated K-fold cross-validation procedure for selecting tuning parameters. We designed the cross-validation procedure to select the tuning parameter that yields highest sNB when weighted recalibration is employed. An important note is that in order to implement this cross-validation procedure, at least either δ or λ must be pre-specified, as well as $[R_l, R_u]$. As discussed in section B.2, the choice of which parameter is pre-specified depends on the desired weight function form. For convenience, we describe the procedure when an exponential decay type weight function is desired (as in Figure 2A of main text), and therefore δ is fixed *a priori* and tuning is conducted to select λ. The procedure is equivalent if a step weight function is desired (as in Figure 2B of main text), where λ is fixed and a cross-validation is used to select δ.

I: Obtain grid of tuning parameters
As described in section 2.3, tuning parameters δ and λ do not have clear clinical interpretation. Instead, a range of suitable RAW values can be used to obtain a grid of λ, (e.g. RAW $\in [0.1, 0.2, \ldots, 0.9]$). Given values of $[R_l, R_u]$, δ, and RAW, solve for λ using equation (7), using `uniroot` function in `R`. Repeat for all values of RAW values to obtain a grid of λ values.

II: Implement single round of K-fold cross-validation
Once a range of tuning parameters is obtained, randomly partition data into K equal folds. Calculate the estimated sNB of λ (δ) within fold k, $sNB_{k,m}(\lambda)$. Repeat for all K folds, and define

$$cv.sNB_m(\lambda) = \frac{1}{K}\sum_{i=k}^{K} cv.sNB_{k,m}(\lambda)$$

to be the cross-validated estimate of sNB from one round of K-fold cross-validation for tuning parameter λ. This process is repeated for the grid of λ values obtained in step I.

III: Repeat K-fold cross-validation M times
Since a single round of cross-validation can be noisy, we propose that cross-validation be repeated multiple times with independent random partitions and the results be averaged. Repeat step II M times, with a new random partitioning each time. Define

$$cv.sNB(\lambda) = \frac{1}{M}\sum_{m=1}^{M} cv.sNB_m(\lambda)$$

to be the repeated cross-validated estimate of sNB for tuning parameter λ. We used $cv.sNB(\lambda)$ to denote the average cross-validated estimate of sNB for tuning parameter λ. We have found that 25 replications of 5-fold cross-validation leads to a suitable reduction in noise.

IV: Compute cross-validated standard error and apply "one-standard error rule"
Following Friedman et al.[4] we implement a "one-standard error" rule for tuning parameter selection. This provides protection against overfitting the data and selecting a tuning parameter that is too extreme. The one-standard error rule also ensures that for instances when weighted logistic recalibration does not provide any gains in sNB beyond standard logistic recalibration, the largest tuning parameter is chosen, and the weighted logistic solution approximates the standard logistic recalibration solution. Under the "one-standard error" rule the selected tuning parameter

$$\lambda = \max\{\lambda : cv.sNB(\lambda) \leq cv.sNB(\lambda^*) - \hat{\sigma}(cv.sNB(\lambda^*))\},$$

where λ^* is the tuning parameter with largest $cv.sNB(\lambda)$ and $\hat{\sigma}(cv.sNB(\lambda^*))$ is the corresponding standard error of λ^*. That is, we propose to select the largest tuning parameter (and therefore apply less down-weighting) such that the cross-validated sNB of that tuning parameter is still within one standard error of the tuning parameter with highest observed $cv.sNB(\lambda)$.

To employ the one-standard error rule, we must estimate the standard error of $cv.sNB(\lambda)$, which we will refer to as $\sigma(cv.sNB(\lambda))$. The calculation of $\sigma(cv.sNB(\lambda))$ requires accounting for the dependence between estimates of $cv.sNB_m(\lambda)$. We can therefore think of the variance of $cv.sNB(\lambda)$ as being composed of two parts: (1) the variation in sNB from a single round of K-fold cross-validation (i.e. $\text{var}[cv.sNB_m(\lambda)]$), which comprises the "within" repetition variance, and (2) the variation between each of the M estimates of estimates (i.e. $\text{var}[cv.sNB_1(\lambda), cv.sNB_2(\lambda), \ldots, cv.sNB_M(\lambda)]$), which we refer to as the "between" repetition variation.

Gelman (1992) give a variance estimator of convergence diagnostic statistic used when Markov Chain Monte Carlo with multiple chains are performed.[5] The variance estimator accounts for both the variability of the statistic "within" a single chain, and the variance of the statistic across, or "between", chains. Analogously, we can use this framework to estimate the "within" repetition variance and the "between" repetition variance. We adapt the procedure presented by Gelman (1992) to obtain a variance estimate of $\sigma(cv.sNB)$. We denote the "within" repetition variance as W and the "between" repetition variance as B.

The "within" repetition is the mean of $var(cv.sNB_m(\lambda))$, across the M repetitions,

$$W(\lambda) = \frac{1}{M}\sum_{m=1}^{M} \text{var}(cv.sNB_m(\lambda)) \tag{13}$$

$$= \frac{1}{M}\sum_{m=1}^{M} \frac{1}{K-1}\sum_{k=1}^{K}(cv.sNB_{k,m}(\lambda) - cv.sNB_m(\lambda))^2, \tag{14}$$

$$\tag{15}$$

Following Gelman, we define the "between" repetition variance to be

$$B(\lambda) = K\text{var}[cv.sNB_1(\lambda), cv.sNB_2(\lambda), \ldots, cv.sNB_M(\lambda)]$$
$$= \frac{K}{M-1}\sum_{m=1}^{M}\left[cv.sNB_m(\lambda) - cv.sNB(\lambda)\right]^2,$$

where the K multiplier accounts for dependence between each $cv.sNB_m(\lambda)$ and fold size. Note that if K is small and there will be less variability in the estimates of $cv.sNB_m$ across replications, since the each round of cross-validation can produce relatively coarse partitions. Comparatively, if K is large the variability may increase due increased likelihood of observing unique partitions across the M replications.

Combining these two piece give a heuristic estimate of the cross-validation variance. We augment the formula slightly from that given in Gelman (1992) to account for the fact that as the number of cross-validation repetitions increases, the between-repetition variability should decrease, since the likelihood of sampling the same fold partition will increase as repetitions increase. The proposed estimator cross-validation standard error is

$$\hat{\sigma}(cv.sNB(\lambda)) = \sqrt{\frac{K-1}{K}W(\lambda) + \frac{1}{M}B(\lambda)}. \tag{16}$$

C Additional Simulation Results

Table 1 shows the hyper-parameters used to simulate risks for each scenario considered. Figures 1 - 4 show the piece-wise polynomial functions used to induce miscalibration for each simulation example.

Figures 5-8 give additional information about each example, including the distribution of predicted risks and calibration curves. Tables 2-5 give detailed numerical results. Finally, Table 6 summarizes results for each example across 500 repeated simulations, showing the proposed methods produce higher average sNB with comparable variability compared to standard logistic recalibration.

Table 1: Hyperparameters for Beta distribution used for generating true risks (p_i) in simulation examples

**Subpopulation**	b_m	$E[p_i]$	α_m	β_m
		**Example 1**		
Subpop. 1	0.33	0.03	0.6	19.4
Subpop. 2	0.34	0.05	0.5	9.5
Subpop. 3	0.33	0.5	4	4
		**Example 2**		
Subpop. 1	0.34	0.05	0.5	9.5
Subpop. 2	0.33	0.15	1	8.5
Subpop. 3	0.33	0.5	1	1
		**Example 3**		
Subpop. 1	0.34	0.05	1	19
Subpop. 2	0.33	0.15	1.5	8.5
Subpop. 3	0.33	0.5	1	1
		**Example 4**		
Subpop. 1	0.4	0.05	1	19
Subpop. 2	0.4	0.1	2	18
Subpop. 3	0.2	0.5	1	1

$$f_1(p_i) = \begin{cases} 0.3(p+0.003)^{0.26} - 0.05 & : p_i \in [0, 0.1) \\ 4.5p^{4.37} + 0.116 & : p_i \in [0.1, 0.6) \\ -0.2p^{-2.15} + 1.2 & : p_i \in [0.6, 1] \end{cases}$$

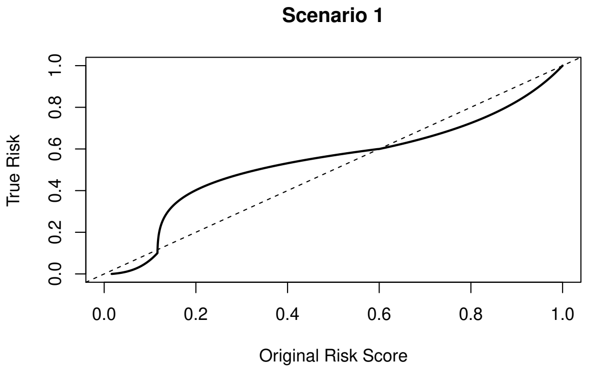

Figure 1: Miscalibration setting for simulation example 1. Moderate risks are underestimated, while high and low risks are overestimated

$$f_2(p_i) = \begin{cases} 0.3p^{0.8} & : p_i \in [0, 0.12) \\ 0.6p^{2.2} + 0.05 & : p_i \in [0.12, 0.46) \\ 1.7(p - 0.4)^{0.4} - 0.4 & : p_i \in [0.46, 1] \end{cases}$$

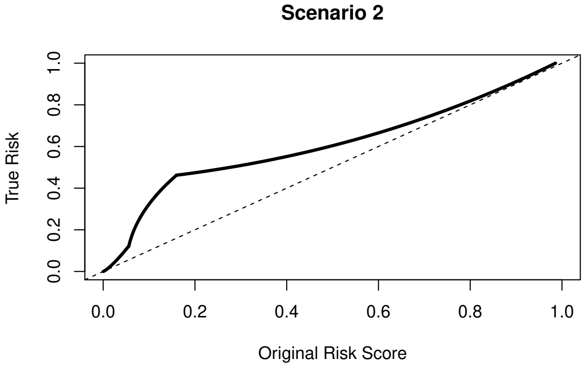

Figure 2: Miscalibration setting for simulation example 2. All risk are underestimated, with moderate risks having worse miscalibration

$$f_3(p_i) = \begin{cases} 139p_i^{3.2} & : p_i \in [0, 0.1) \\ -0.1p_i^{-0.83} - 0.1 & : p_i \in [0.1, 0.54) \\ 0.4p_i^{2.5} + 0.5 & : p_i \in [0.54, 1] \end{cases}$$

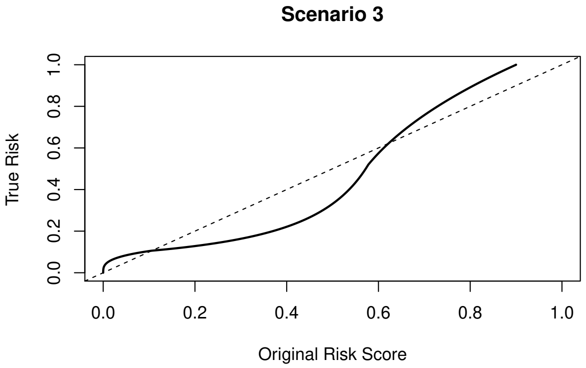

Figure 3: Miscalibration setting for simulaion example 3. Moderate risk scores are overestimated, while low and high risk scores tend towards underestimation.

$$f_4(p_i) = \begin{cases} 139p_i^{3.2} + 0.07 & : p_i \in [0, 0.15) \\ -0.1p_i^{-0.83} + 0.869 & : p_i \in [0.15, 0.54) \\ 0.38p_i^2.5 + 0.62 & : p_i \in [0.54, 1] \end{cases}$$

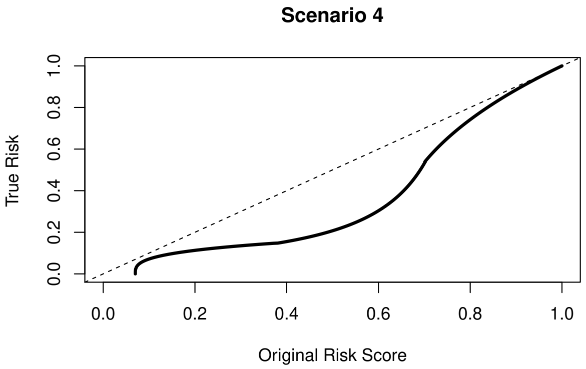

Figure 4: Miscalibration setting for simulation example 4. All risk are overestimated, with moderate risks having worse miscalibration

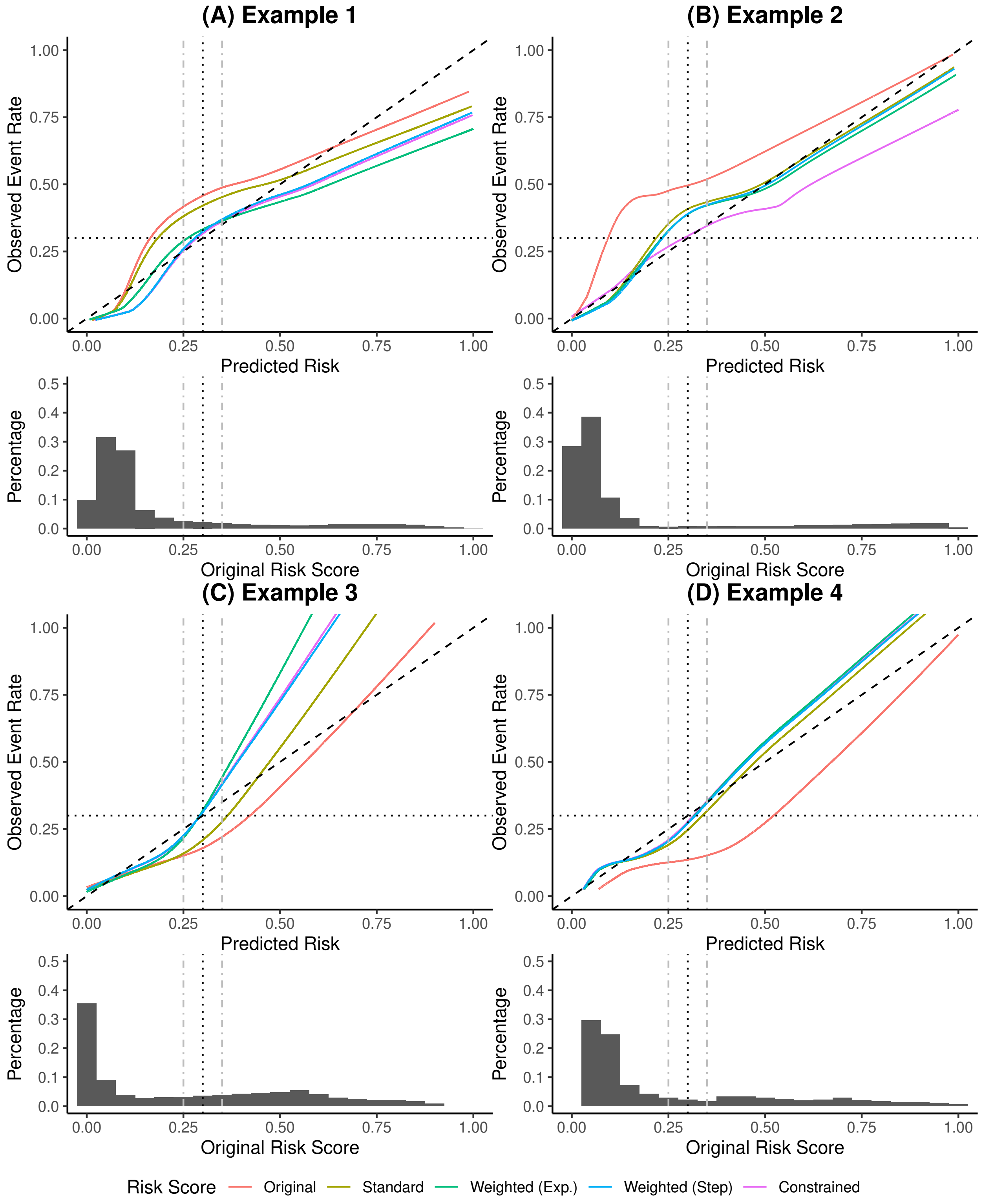

Figure 5: Calibration curves and risk score distribution for simulation examples with sample size $N = 10000$. Calibration curves for the original, standard recalibrated, weighted recalibrated, and constrained recalibrated risk models are show for each simulation example setting. The histogram of the original simulated risk score is also presented. Dotted lines indicate the clinically relevant risk threshold, $R = 0.3$. The dashed is the identity line.

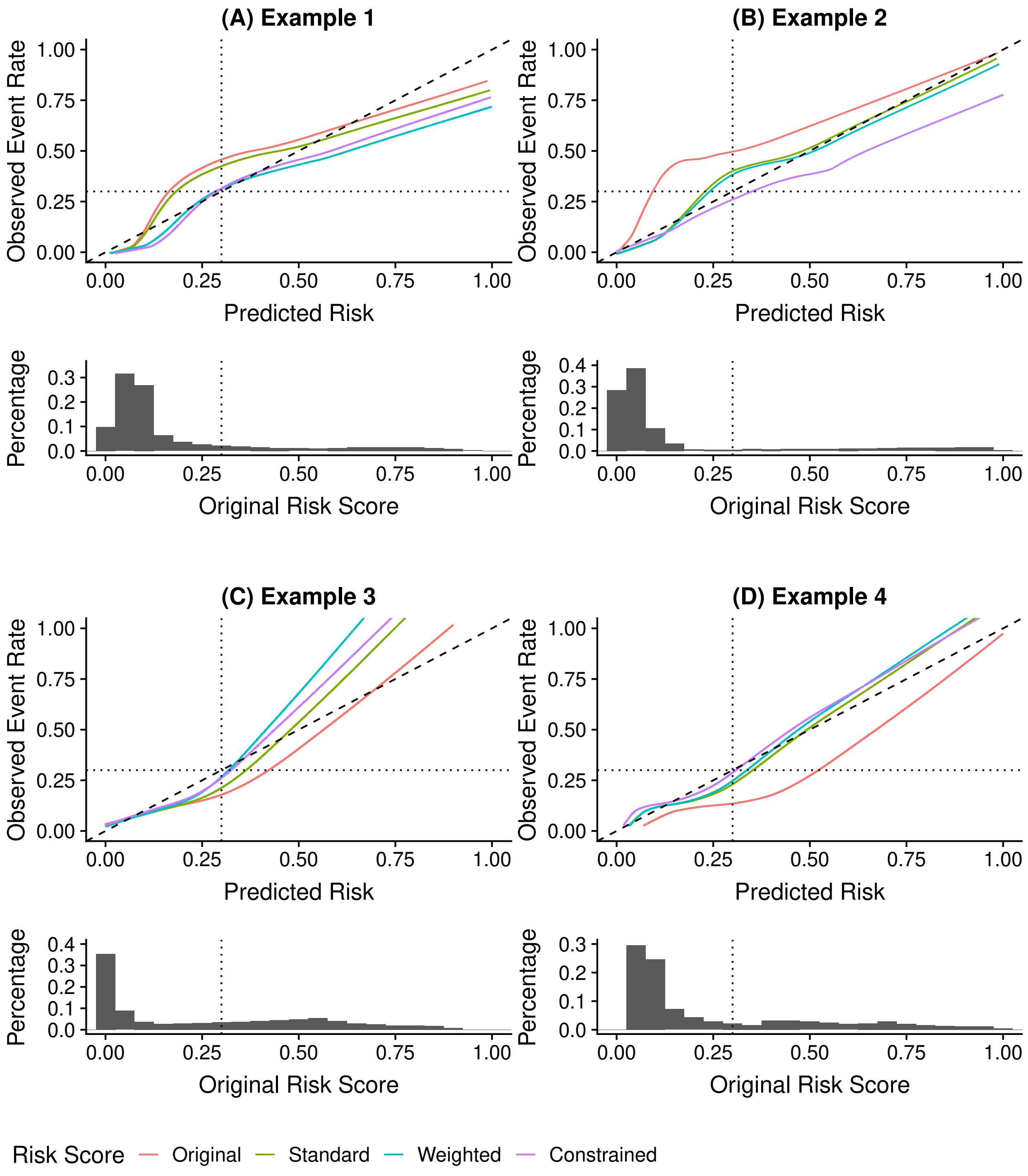

Figure 6: Calibration curves and risk score distribution for simulation examples with sample size $N = 5000$. Calibration curves for the original, standard recalibrated, weighted recalibrated, and constrained recalibrated risk models are show for each simulation example setting. The histogram of the original simulated risk score is also presented. Dotted lines indicate the clinically relevant risk threshold, $R = 0.3$. The dashed is the identity line.

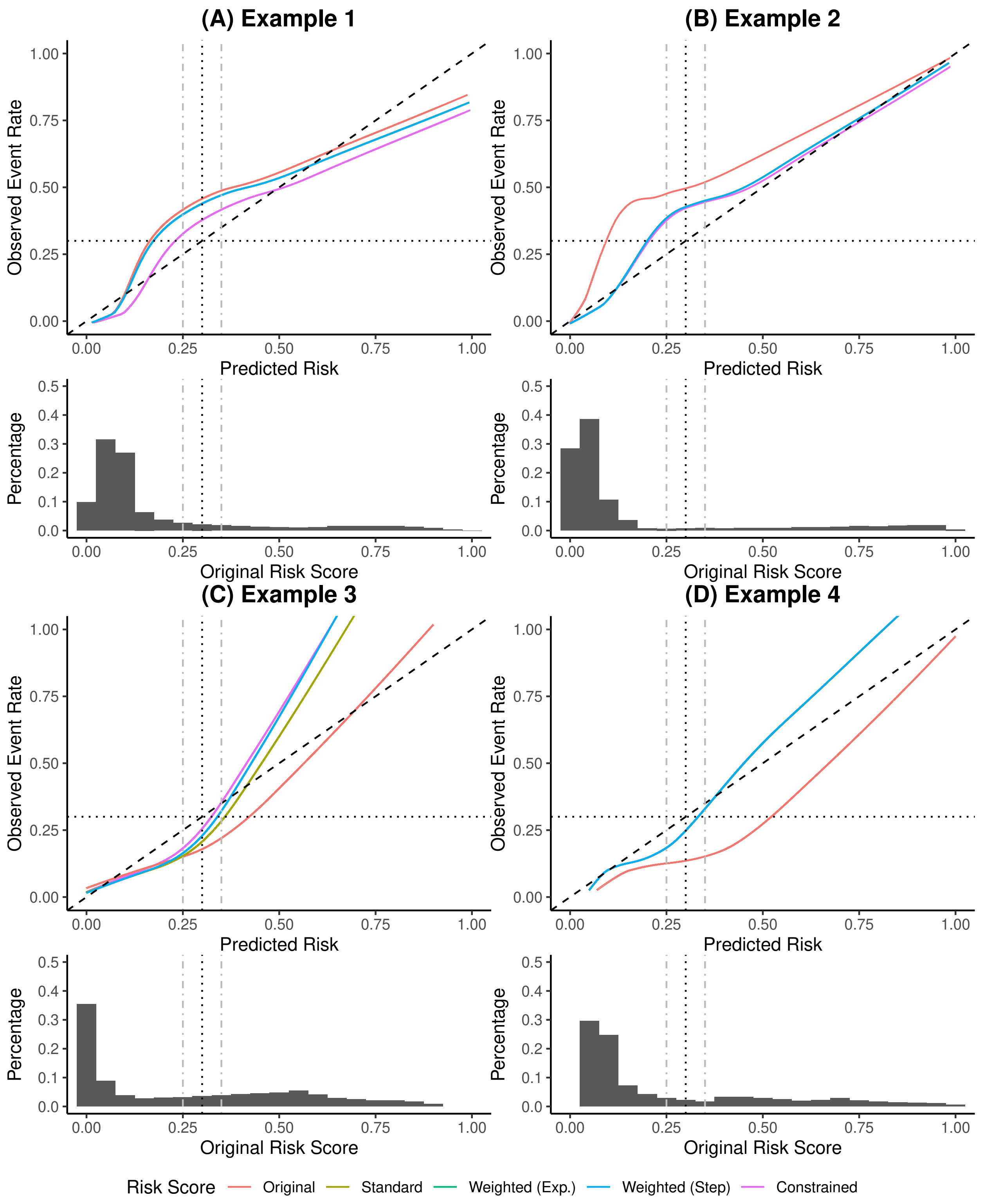

Figure 7: Calibration curves and risk score distribution for simulation examples with sample size $N = 1000$. Calibration curves for the original, standard recalibrated, weighted recalibrated, and constrained recalibrated risk models are show for each simulation example setting. The histogram of the original simulated risk score is also presented. Dotted lines indicate the clinically relevant risk threshold, $R = 0.3$. The dashed is the identity line.

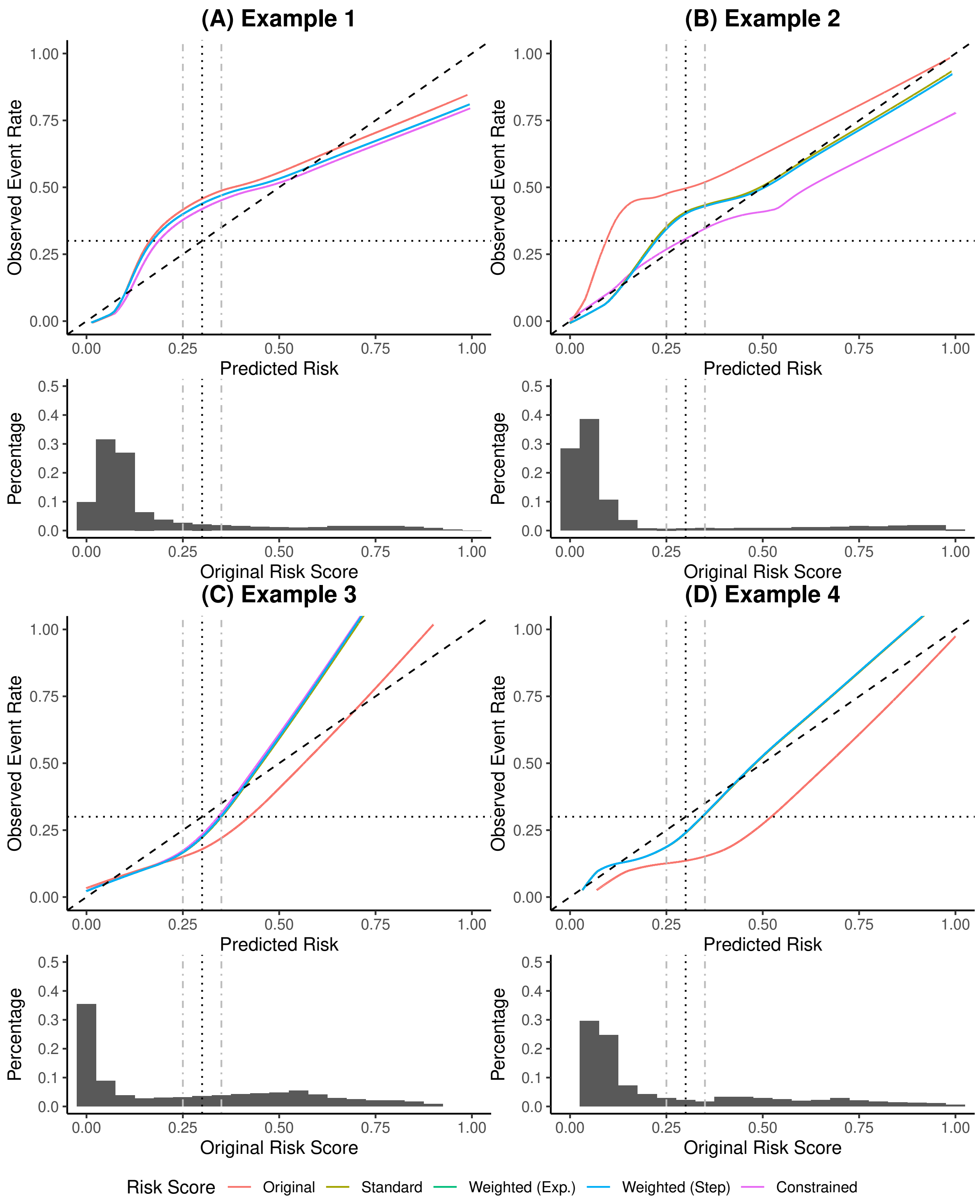

Figure 8: Calibration curves and risk score distribution for simulation examples with sample size $N = 500$. Calibration curves for the original, standard recalibrated, weighted recalibrated, and constrained recalibrated risk models are show for each simulation example setting. The histogram of the original simulated risk score is also presented. Dotted lines indicate the clinically relevant risk threshold, $R = 0.3$. The dashed is the identity line.

Table 2: Performance of standard, weighted (using exponential form and step form weights), and constrained logistic recalibration in Example 1.

**Measure**	**Orig. (No Recal.)**	**Std. Recal.**	**Wt. Recal.** Exponential Wt.	**Wt. Recal.** Step Wt.	**Const. Recal.**
			**Sample Size n=10,000**		
sNB	0.430	0.457	0.499	0.499	0.501
TPR	0.581	0.634	0.745	0.744	0.751
FPR	0.083	0.097	0.135	0.135	0.138
$(\hat{\alpha}_0, \hat{\alpha}_1)$	-	(0.40, 1.16)	(1.60, 1.59)	(0.89, 1.13)	(0.96, 1.15)
Prop. Assigned Intervention	0.18	0.20	0.25	0.25	0.26
CV-Selected RAW (λ)	-	-	0.24 ($\lambda = 0.034$)	0.06 ($\delta = 0.06$)	-
Effective Sample Proportion (%)	100	100	26.1	9.39	100
			**Sample Size n=5,000**		
sNB	0.430	0.455	0.503	0.500	0.504
TPR	0.581	0.629	0.759	0..750	0.761
FPR	0.083	0.096	0.140	0.137	0.141
Prop. Assigned Intervention	0.18	0.20	0.26	0.25	0.26
$(\hat{\alpha}_0, \hat{\alpha}_1)$	-	(0.34, 1.12)	(1.46, 1.45)	(0.86, 1.09)	(0.96, 1.12)
CV-Selected RAW (λ)	-	-	0.18 ($\lambda = 0.024$)	0.8 ($\delta = 0.8$)	-
Effective Sample Proportion (%)	100	100	21.0	12.4	100
			**Sample Size n=1,000**		
sNB	0.430	0.445	0.445	0.445	0.481
TPR	0.581	0.608	0.609	0.609	0.688
FPR	0.083	0.090	0.090	0.090	0.114
$(\hat{\alpha}_0, \hat{\alpha}_1)$	-	(0.19, 1.07)	(0.19, 1.07)	(0.19, 1.07)	(0.57, 1.08)
Prop. Assigned Intervention	0.18	0.19	0.19	0.19	0.22
CV-Selected RAW (λ)	-	-	0.99 ($\lambda = 6.893$)	0.99 ($\delta = 0.99$)	-
Effective Sample Proportion (%)	100	100	99.9	99.0	100
			**Sample Size n=500**		
sNB	0.430	0.446	0.446	0.446	0.458
TPR	0.581	0.610	0.610	0.610	0.636
FPR	0.083	0.090	0.090	0.090	0.098
$(\hat{\alpha}_0, \hat{\alpha}_1)$	-	(0.22, 1.10)	(0.22, 1.10)	(0.23, 1.10)	(0.36, 1.11)
Prop. Assigned Intervention	0.16	0.18	0.18	0.19	0.20
CV-Selected RAW (λ)	-	-	0.99 ($\lambda = 6.442$)	0.99 ($\delta = 0.99$)	-
Effective Sample Proportion (%)	100	100	99.0	99.0	100

Table 3: Performance of standard, weighted (using exponential form and step form weights), and constrained logistic recalibration in Example 2.

**Measure**	**Orig. (No Recal.)**	**Std. Recal.**	**Wt. Recal.** Exponential Wt.	**Wt. Recal.** Step Wt.	**Const. Recal.**
			**Sample Size n=10,000**		
sNB	0.475	0.511	0.521	0.518	0.525
TPR	0.546	0.615	0.640	0.636	0.650
FPR	0.052	0.077	0.088	0.086	0.092
$(\hat{\alpha}_0, \hat{\alpha}_1)$	-	(0.75, 0.89)	(0.99, 0.96)	(0.826, 0.883)	(0.97, 0.90)
Prop. Assigned Intervention	0.17	0.21	0.22	0.22	0.23
CV-Selected RAW (λ)	-	-	0.70 ($\lambda = 0.195$)	0.29 (δ= 0.29)	-
Effective Sample Proportion (%)	100	100	71.6	33.5	100
			**Sample Size n=5,000**		
sNB	0.475	0.511	0.523	0.519	0.524
TPR	0.546	0.619	0.644	0.637	0.647
FPR	0.052	0.080	0.089	0.087	0.091
$(\hat{\alpha}_0, \hat{\alpha}_1)$	-	(0.64, 0.82)	(0.86, 0.88)	(0.70, 0.81)	(0.75, 0.82)
Prop. Assigned Intervention	0.17	0.21	0.22	0.22	0.22
CV-Selected RAW (λ)	-	-	0.74 ($\lambda = 0.214$)	0.33 ($\delta = 0.33$)	-
Effective Sample Proportion (%)	100	100	75.2	36.7	100
			**Sample Size n=1,000**		
sNB	0.475	0.491	0.491	0.491	0.491
TPR	0.546	0.581	0.581	0.581	0.581
FPR	0.052	0.066	0.066	0.066	0.066
$(\hat{\alpha}_0, \hat{\alpha}_1)$	-	(0.51, 0.84)	(0.52, 0.84)	(0.52, 0.84)	(0.51, 0.83)
Prop. Assigned Intervention	0.17	0.19	0.19	0.19	0.19
CV-Selected RAW (λ)	-	-	0.99 ($\lambda = 7.557$)	0.99 ($\delta = 0.99$)	-
Effective Sample Proportion (%)	100	100	99.0	99.1	100
			**Sample Size n=500**		
sNB	0.475	0.510	0.513	0.511	0.524
TPR	0.546	0.616	0.624	0.617	0.647
FPR	0.052	0.077	0.082	0.077	0.091
$(\hat{\alpha}_0, \hat{\alpha}_1)$	-	(0.77, 0.90)	(0.86, 0.93)	(0.77, 0.90)	(0.99, 0.94)
Prop. Assigned Intervention	0.17	0.21	0.21	0.21	0.22
CV-Selected RAW (λ)	-	-	0.90 ($\lambda = 0.775$)	0.99 ($\delta = 0.99$)	-
Effective Sample Proportion (%)	100	100	90.5	99	100

Table 4: Performance of standard, weighted (using exponential form and step form weights), and constrained logistic recalibration in Example 3.

**Measure**	**Orig. (No Recal.)**	**Std. Recal.**	**Wt. Recal.** Exponential Wt.	**Wt. Recal.** Step Wt.	**Const. Recal.**
			**Sample Size n=10,000**		
sNB	0.440	0.449	0.502	0.503	0.503
TPR	0.844	0.836	0.757	0.751	0.751
FPR	0.282	0.271	0.173	0.173	0.178
$(\hat{\alpha}_0, \hat{\alpha}_1)$	-	(-0.31, 0.69)	(-0.69, 0.53)	(-0.663, 0.684)	(-0.66, 0.66)
Prop. Assigned Intervention	0.41	0.40	0.32	0.31	0.31
CV-Selected RAW (λ/δ)	-	-	0.57 ($\lambda = 0.096$)	0.11 ($\delta = 0.11$)	-
Effective Sample Proportion (%)	100	100	59.6	17.4	100
			**Sample Size n=5,000**		
sNB	0.440	0.458	0.484	0.480	0.488
TPR	0.844	0.830	0.795	0.801	0.789
FPR	0.282	0.260	0.217	0.224	0.210
$(\hat{\alpha}_0, \hat{\alpha}_1)$	-	(-0.30, 0.76)	(-0.54, 0.64)	(-0.45, 0.76)	(-0.49, 0.79)
Prop. Assigned Intervention	0.41	0.39	0.35	0.36	0.34
CV-Selected RAW (λ/δ)	-	-	0.73 ($\lambda = 0.200$)	0.22 ($\delta = 0.22$)	-
Effective Sample Proportion (%)	100	100	74.5	27.3	100
			**Sample Size n=1,000**		
sNB	0.440	0.440	0.457	0.440	0.476
TPR	0.844	0.844	0.831	0.844	0.808
FPR	0.282	0.282	0.262	0.282	0.232
$(\hat{\alpha}_0, \hat{\alpha}_1)$	-	(-0.36, 0.57)	(-0.46, 0.54)	(-0.36, 0.58)	(-0.52, 0.58)
Prop. Assigned Intervention	0.41	0.41	0.39	0.41	0.36
CV-Selected RAW (λ/δ)	-	-	0.89 ($\lambda = 0.526$)	0.99 (δ =0.99)	-
Effective Sample Proportion (%)	100	100	89.8	99.1	100
			**Sample Size n=500**		
sNB	0.440	0.459	0.461	0.460	0.475
TPR	0.844	0.828	0.827	0.828	0.810
FPR	0.282	0.258	0.256	0.257	0.232
$(\hat{\alpha}_0, \hat{\alpha}_1)$	-	(-0.38, 0.66)	(-0.39, 0.65)	(-0.38, 0.66)	(-0.49, 0.63)
Prop. Assigned Intervention	0.41	0.39	0.39	0.39	0.36
CV-Selected RAW (λ/δ)	-	-	0.99 ($\lambda = 6.211$)	0.99 ($\delta = 0.99$)	-
Effective Sample Proportion (%)	100	100	99.1	99.1	100

Table 5: Performance of standard, weighted (using exponential form and step form weights), and constrained logistic recalibration in Example 4.

**Measure**	**Orig. (No Recal.)**	**Std. Recal.**	**Wt. Recal.** Exponential Wt.	**Wt. Recal.** Step Wt.	**Const. Recal.**
		**Sample Size n=10,000**			
sNB	0.282	0.422	0.430	0.430	0.431
TPR	0.759	0.641	0.622	0.622	0.618
FPR	0.212	0.097	0.086	0.086	0.083
$(\hat{\alpha}_0, \hat{\alpha}_1)$	-	(-0.90, 0.97)	(-0.98, 0.92)	(-0.989, 0.954)	(-1.01, 0.95)
Prop. Assigned Intervention	0.30	0.18	0.17	0.17	0.17
CV-Selected RAW (λ)	-	-	0.76 ($\lambda = 0.212$)	0.38 ($\delta = 0.38$)	-
Effective Sample Proportion (%)	100	100	76.7	40.4	100
		**Sample Size n=5,000**			
sNB	0.282	0.413	0.421	0.425	0.435
TPR	0.759	0.652	0.641	0.638	0.605
FPR	0.212	0.106	0.098	0.095	0.076
$(\hat{\alpha}_0, \hat{\alpha}_1)$	-	(-0.84, 0.99)	(-0.90, 0.95)	(-0.92, 0.97)	(-1.11, 1.11)
Prop. Assigned Intervention	0.30	0.19	0.19	0.18	0.16
CV-Selected RAW (λ)	-	-	0.86 ($\lambda = 0.413$)	0.45 (δ 0.45) -	
Effective Sample Proportion (%)	100	100	86.4	47.1	100
		**Sample Size n=1,000**			
sNB	0.282	0.417	0.417	0.417	0.417
TPR	0.759	0.647	0.647	0.646	0.647
FPR	0.212	0.102	0.102	0.102	0.102
$(\hat{\alpha}_0, \hat{\alpha}_1)$	-	(-0.87, 0.81)	(-0.87, 0.81)	(-0.87, 0.81)	(-0.87, 0.81)
Prop. Assigned Intervention	0.30	0.19	0.19	0.19	0.19
CV-Selected RAW (λ)	-	-	0.99 ($\lambda = 5.101$)	0.99 ($\delta = 0.99$)	-
Effective Sample Proportion (%)	100	100	99.0	99.0	100
		**Sample Size n=500**			
sNB	0.282	0.417	0.418	0.418	0.417
TPR	0.759	0.646	0.645	0.646	0.646
FPR	0.212	0.102	0.101	0.102	0.102
$(\hat{\alpha}_0, \hat{\alpha}_1)$	-	(-0.87, 0.97)	(-0.88, 0.97)	(-0.87, 0.97)	(-0.87, 0.97)
Prop. Assigned Intervention	0.30	0.19	0.19	0.19	0.19
CV-Selected RAW (λ)	-	-	0.99 ($\lambda = 6.414$)	0.99 ($\delta = 0.99$)	-
Effective Sample Proportion (%)	100	100	99.0	99.0	100

Table 6: Summary of sNB obtained after recalibration across 500 repeated simulations. Mean (SD) of sNB of risk score after standard, weighted (with exponential and step weights), and constrained recalibration. Recalibration parameters are estimated in a training dataset with 1000 observations and sNB is evaluated in an independent dataset 10^6 observations.

**Example**	**Standard**	**Weighted Exp.**	**Weighted Step**	**Constrained**
Example 1	0.478 (0.009)	0.479 (0.009)	0.478 (0.009)	0.484 (0.012)
Example 2	0.466 (0.016)	0.476 (0.017)	0.474 (0.018)	0.484 (0.018)
Example 3	0.463 (0.015)	0.472 (0.018)	0.470 (0.018)	0.482 (0.019)
Example 4	0.383 (0.021)	0.392 (0.020)	0.388 (0.021)	0.401 (0.021)

References

[1] Baker SG, Van Calster B, Steyerberg EW. Evaluating a new marker for risk prediction using the test tradeoff: an update. The International Journal of Biostatistics. 2012;8(1):1–37.

[2] Pepe MS. The Statistical Evaluation of Medical Tests for Classification and Prediction. Oxford University Press; 2003.

[3] Baker SG, Cook NR, Vickers A, Kramer BS. Using relative utility curves to evaluate risk prediction. Journal of the Royal Statistical Society: Series A (Statistics in Society). 2009;172(4):729–748.

[4] Friedman J, Hastie T, Tibshirani R. The elements of statistical learning. vol. 1. Springer series in statistics New York, NY, USA; 2001.

[5] Gelman A, Rubin DB, et al. Inference from iterative simulation using multiple sequences. Statistical Science. 1992;7(4):457–472.
